# PhenQE8, a Novel Ligand of the Human Telomeric Quadruplex †

**DOI:** 10.3390/ijms22020749

**Published:** 2021-01-13

**Authors:** Patricia B. Gratal, Julia G. Quero, Adrián Pérez-Redondo, Zoila Gándara, Lourdes Gude

**Affiliations:** Departamento de Química Orgánica y Química Inorgánica, Instituto de Investigación Química “Andrés M. del Río” (IQAR), Universidad de Alcalá, 28805 Alcalá de Henares, Madrid, Spain; patricia.gratal@uah.es (P.B.G.); julia.gonzalezq@uah.es (J.G.Q.); adrian.perez@uah.es (A.P.-R.)

**Keywords:** 1,10-phenanthroline, guanyl hydrazones, human telomeric quadruplex, DNA interactions, antitumor agents

## Abstract

A novel quadruplex ligand based on 1,10-phenanthroline and incorporating two guanyl hydrazone functionalities, PhenQE8, is reported herein. Synthetic access was gained in a two-step procedure with an overall yield of 61%. X-ray diffraction studies revealed that PhenQE8 can adopt an extended conformation that may be optimal to favor recognition of quadruplex DNA. DNA interactions with polymorphic G-quadruplex telomeric structures were studied by different techniques, such as Fluorescence resonance energy transfer (FRET) DNA melting assays, circular dichroism and equilibrium dialysis. Our results reveal that the novel ligand PhenQE8 can efficiently recognize the hybrid quadruplex structures of the human telomeric DNA, with high binding affinity and quadruplex/duplex selectivity. Moreover, the compound shows significant cytotoxic activity against a selected panel of cultured tumor cells (PC-3, HeLa and MCF-7), whereas its cytotoxicity is considerably lower in healthy human cells (HFF-1 and RPWE-1).

**Highlights**

- A novel G-quadruplex DNA ligand, PhenQE8, based on 1,10-phenanthroline and containing two guanyl hydrazone groups was synthesized with good yields.
- PhenQE8 can adopt an extended conformation with one molecular dimension expanding ~16 Å.
- PhenQE8 can recognize the hybrid (mixed) structures of the telomeric quadruplex with good binding affinity.
- PhenQE8 shows high quadruplex/duplex DNA binding selectivity.
- PhenQE8 exhibits antitumor activity in cell culture and presents lower cytotoxicity in healthy human fibroblasts and normal prostate cells.

## 1. Introduction

From the variety of noncanonical secondary structures that DNA can adopt, quadruplexes have been extensively studied and have aroused an increasing interest over the last decades [1,2,3]. G-quadruplexes typically occur in guanine-rich genomic regions with the association of the guanine residues in the form of tetrads. The tetrads are flat and cyclic, and each one is constituted by four guanine bases that interact with each other through a total of eight Hoogsteen hydrogen bonds. Two or more guanine tetrads can then associate via π-π stacking interactions to form the quadruplex tridimensional structure, which is additionally stabilized by the presence of metal cations in its central channel, usually, Na^+^ or K^+^. G-quadruplexes have been found in several genomic regions with relevant biological functions: in telomeres [4], in the origins of replication [5], the 5′-UTR regions [6], and in certain oncogenic promoters (c-myc, c-kit, k-ras, etc.), to name just a few [7]. Moreover, it is commonly accepted that quadruplexes are especially abundant in tumor cells and, in addition, their implication in epigenetic regulation is currently addressing considerable attention [8,9]. Therefore, G-quadruplexes constitute interesting targets in the development of new and efficient therapeutics, with a special focus on those that can find applications in the treatment of cancer [10,11].

G-quadruplexes are polymorphic in nature, mostly because the way the quadruplex structure folds itself is clearly dependent on several factors and on the experimental conditions, including type of ion and concentration [12,13], sequence, temperature, etc. and because of their intrinsically highly dynamic character [14]. Therefore, many different and biologically relevant quadruplex structures have been characterized and reported over the last years and, in particular, the human telomeric structure polymorphism, and the effects observed upon ligand binding, have been the object of intense investigation [15,16,17,18,19,20]. The telomeric sequence displays a pronounced polymorphism and several structures have been characterized, predominantly the hybrid mixture and antiparallel and parallel topologies [21,22,23,24]. Moreover, this sequence is closely related to the catalytic activity of ribonucleoprotein telomerase, abnormally significant in many types of human tumors [25]. Therefore, both telomeres and telomerase have become interesting targets for the discovery of novel anticancer agents [26]. In particular, the use of small-molecule ligands as potential telomere targeting agents that can stabilize the G-quadruplex structures at the end of the chromosomes has drawn considerable attention in the last years [27,28]. 

Regarding the principles that sustain the rational design of G-quadruplex stabilizing ligands, there are several common structural features that have been found to be present in some of the best quadruplex ligands studied thus far [29,30,31,32]. Some that deserve mention are: (a) a delocalized π-system with an adequate size and surface to stabilize the G-quadruplex by stacking interactions with the terminal tetrads; (b) a positive charge or electronic deficiency that can be located in close proximity to the center of G-quadruplex, simulating the stabilizing effect of K^+^ or Na^+^ ions and (c) the presence of at least one flexible side chain with positive charge at physiological pH, which can provide additional interactions with the grooves and/or loops of the quadruplex. Taking into account these considerations, different types of molecules have been designed, synthesized and evaluated, which can be categorized in three main families: fused polyaromatic systems, macrocyclic compounds (either of natural or synthetic origin) and nonfused aromatic systems. Without being exhaustive [33,34], some examples of compounds studied as quadruplex ligands, and that have shown biological activity, include 2,6-diamidoanthraquinone, bisA, BRACO-19, PIPER, telomestatin, pyridostatin, TmPyP4, NMM, quarfloxacin, RHPS4, CX-5461, 360A, PhenDC3 and naphthalene diimides. Unfortunately, practical clinical applications remain elusive for most of these compounds, with some rare exceptions such as quarfloxacin and CX-5461.

Among the great variety of aromatic heterocycles that have been demonstrated interesting properties as potential quadruplex ligands, the 1,10-phenanthroline heterocyclic system deserves a mention. Several derivatives have been found to bind to quadruplexes with good affinity and selectivity, both in the form of organic ligands [35,36,37,38] or as metallo-organic derivatives [35,39,40,41,42]

On the other hand, natural and synthetic guanidine derivatives have been of great interest due to their pronounced biological activity [43]. Among their properties, it is worth mentioning their use in neurodegenerative disorders as anti-inflammatory agents, as inhibitors of the Na^+^/H^+^ exchanger and blockers of the epithelial sodium channel, as inhibitors of F1F0-ATPase, as cardiovascular and as antidiabetic or chemotherapeutic agents. Moreover, different authors have studied the high affinity of this group when incorporated into molecules that interact with DNA or RNA. In contrast to the amino group, the guanidine moiety has a higher basicity, is flatter and exhibits greater directionality in its H bonding interactions [44]. Luedtke et al. demonstrated the guanidine capacity to interact with G-quadruplex DNA with the synthesis and biological evaluation of guanidinium-modified phthalocyanines [45]. Overall, the guanidine group can be found as a part of the structure of a few quadruplex ligands [36,46], including in the form of a guanyl hydrazone functionality [47,48]. Furthermore, it has been suggested that compounds containing the guanidine group tend to accumulate at the mitochondria, where they can exert important biological actions [49,50]. 

**Figure 1:**
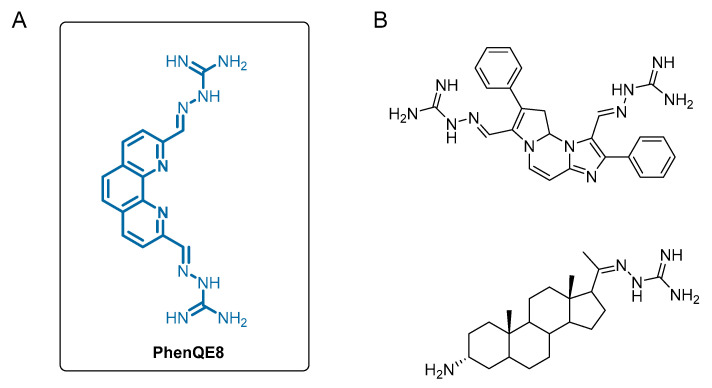
(**A**) Chemical structure of novel G4 ligand PhenQE8; (**B**) Examples of other quadruplex ligands containing the guanyl hydrazone group [47,48].

We are interested in the development of novel phenanthroline derivatives as polycyclic aromatic systems that can promote π-π stacking interactions with DNA quadruplexes. We report herein our recent work on the discovery of a novel telomeric ligand based on 1,10-phenanthroline, designated as PhenQE8 (Figure 1A), incorporating in its structure two-guanyl hydrazone groups as part of the two positively charged side chains, with the aim of increasing interactions with hybrid human telomeric quadruplex structures. To the best of our knowledge there are very few examples of quadruplex ligands that possess the guanyl hydrazone function, with representative examples shown in Figure 1B. We have synthesized PhenQE8 in a simple, two-step sequence of reactions and we have evaluated its binding affinity and quadruplex binding selectivity by competitive and noncompetitive DNA FRET melting assays, circular dichroism, and by competition and noncompetition equilibrium dialysis. Finally, its cytotoxic activity against cultured tumor and healthy cells has been preliminary determined. 

## 2. Results and Discussion

### 2.1. Synthesis and Structural Study of PhenQE8

**Scheme 1:**
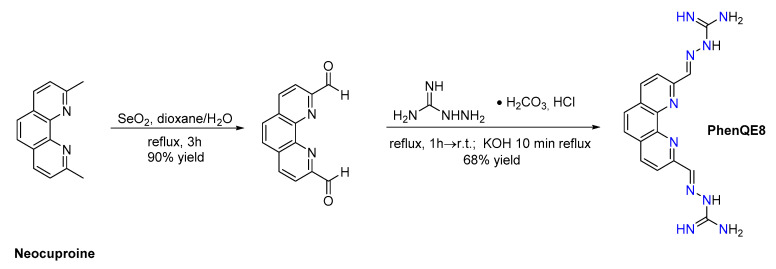
Synthetic route for compound PhenQE8.

The synthesis of compound (2E,2′E)-2,2′-((1,10-phenanthroline-2,9-diyl)bis-(methanylylidene))-bis(hydrazine-1-carboximidamide), designated as PhenQE8, was accomplished following an easy and efficient two-step route as shown in Scheme 1, yielding the desired compound with a 61% global yield. As the starting material, neocuproine (2,9-dimethyl-1,10-phenanthroline) was used, from which oxidation with SeO_2_ led to the intermediate 1,10-phenanthroline-2,9-dicarbaldehyde [51,52], in 90% yield. Subsequently, the dialdehyde was transformed into the corresponding 2,9-bis(guanylhydrazone)-1,10-phenanthroline derivative, PhenQE8, through a condensation reaction with commercial aminoguanidine bicarbonate [53]. The final treatment with strong base at reflux allowed the isolation of the compound, which was expected to be quite basic, as its neutral, nonprotonated form. Treatment with MeOH to filter inorganic salts and subsequent precipitation from a mixture of Et_2_O/MeOH afforded the compound in 68% yield.

The structural study of PhenQE8 in solution was accomplished by ^1^H and ^13^C NMR in (CD_3_)_2_SO and D_2_O, using homonuclear and heteronuclear 2D-NMR techniques for the identification and unambiguous assignment of the proton and carbon resonances. The compound was also characterized by High resolution mass spectrometry (HR-MS) and Fourier-transform infrared (FTIR)-spectroscopy (Methods and Supporting Materials, Figures S1–S11). 

**Figure 2:**
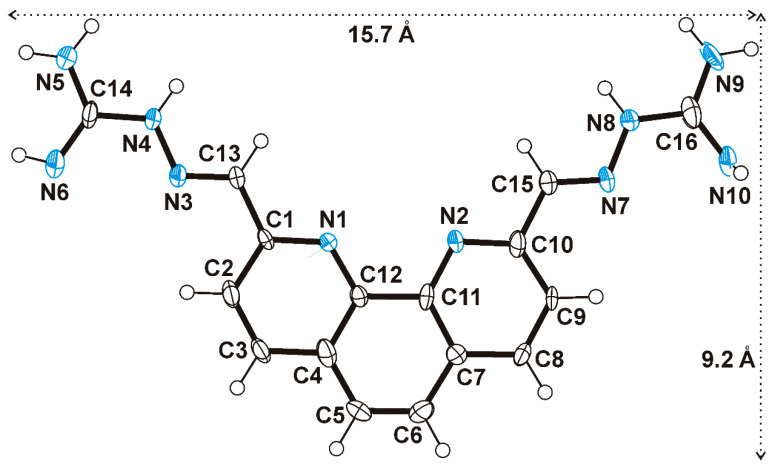
Ortep diagram for one of the two crystallographically independent molecules of PhenQE8. Thermal ellipsoids are drawn at the 50% probability level.

Additionally, PhenQE8 was crystallized from a solution of dimethyl sulfoxide, which allowed further analysis of the structural features in the solid state. Crystals contained two independent molecules (Figure S12) in the asymmetric unit, but there were no significant differences between them. Figure 2 shows one of these independent molecules as an example. The molecule is almost planar with an *E* arrangement for the imine moieties N(3)=C(13) and N(7)=C(15). Considering hydrogen atoms, the dimensions of an imaginary cage in which the molecule is fitted (Figure 2) are 15.7 and 9.2 Å (16.2 and 9.1 Å for the second independent molecule). The molecules of PhenQE8 are associated by π···π interactions with perpendicular distances ranging 3.2–3.4 Å.

### 2.2. Quadruplex and Duplex DNA Interactions

#### 2.2.1. FRET DNA Melting Assays

Initial evaluation of G-quadruplex binding was accomplished on the human telomeric sequence by using variable-temperature (FRET DNA melting) assays, in both noncompetition and competition versions. The telomeric oligonucleotide F21T [54], labeled with the fluorophore FAM at its 5′ end and the fluorophore TAMRA at the 3′ end, was employed, along with oligonucleotide F10T as a comparison for duplex stabilization in parallel noncompetitive experiments [55]. The telomeric quadruplex was folded under potassium-rich ionic conditions, as these are considered similar to the physiological conditions of the cell nucleus. 

**Figure 3:**
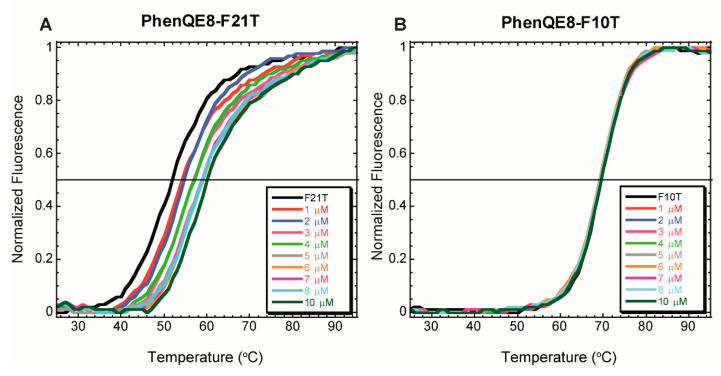
DNA FRET melting experiments for PhenQE8 at a concentration range between 1–10 μM, in the buffer system 10 mM lithium cacodylate, 90 mM LiCl, 10 mM KCl with (**A**) telomeric oligonucleotide F21T; (**B**) duplex DNA F10T.

The degree of quadruplex stabilization as a function of the increase of melting temperature observed upon ligand binding is commonly reported at a reference concentration of 1 μM. Compound PhenQE8, however, exhibited minimal increase in T_m_ at this concentration, but did show a significant effect on T_m_ values at higher concentrations, in a dose-response manner, reaching a ∆T_m_ ~ 7 °C at 5 μM of compound (Figure 3A). This behavior was also observed when the reversed process was analyzed, the folding of Tel22 in the presence of PhenQE8, although the stabilizing effect was slightly lower, as can be appreciated by superimposition of FRET heating and cooling curves and by closer analysis of selected concentration points (Figure S13, panels A and C). 

This result represents the modest ability of PhenQE8 to stabilize the telomeric quadruplex structure. However, when a parallel experiment was run with the labeled DNA duplex sequence F10T, an overlapping of the melting curves was observed with null stabilization of the doubled stranded DNA structure (∆T_m_ ~ 0 °C), even at the highest 10 μM compound concentration (Figure 3B). This indicates a preferential binding and stabilization of the quadruplex structure by PhenQE8, but because F10T displays a higher thermal stability than F21T, these results should be confirmed in additional experiments under real quadruplex versus duplex competition conditions.

Thus, with the aim of assessing quadruplex selectivity, analogous experiments were repeated by maintaining PhenQE8 concentration equal to 5 μM in the absence and in the presence of an unlabeled dsDNA competitive sequence, ds26. This competitor was included in the assays at two different concentrations, 3 and 10 μM, representing a 15-fold and a 50-fold duplex stoichiometric excess, respectively. Subsequently, to estimate the binding selectivity (S), the ∆T_m_ obtained in the presence of the duplex competitor at each concentration versus the ∆T_m_ observed with G4 and ligand only were compared according to the formula that allows the estimation of the S parameter, S = (∆T_m_)_competitor_/(∆T_m_)_w/o competitor_. If the compound under investigation is selective for the quadruplex structure, a slight modification of ∆T_m_ upon addition of a huge excess of ds DNA is expected or, ideally, the value ∆T_m_ should remain unaltered. Consequently, the value of S is commonly used to estimate selectivity of quadruplex ligands, with values closer to 1 representing a high selectivity for the quadruplex DNA structure. 

**Figure 4:**
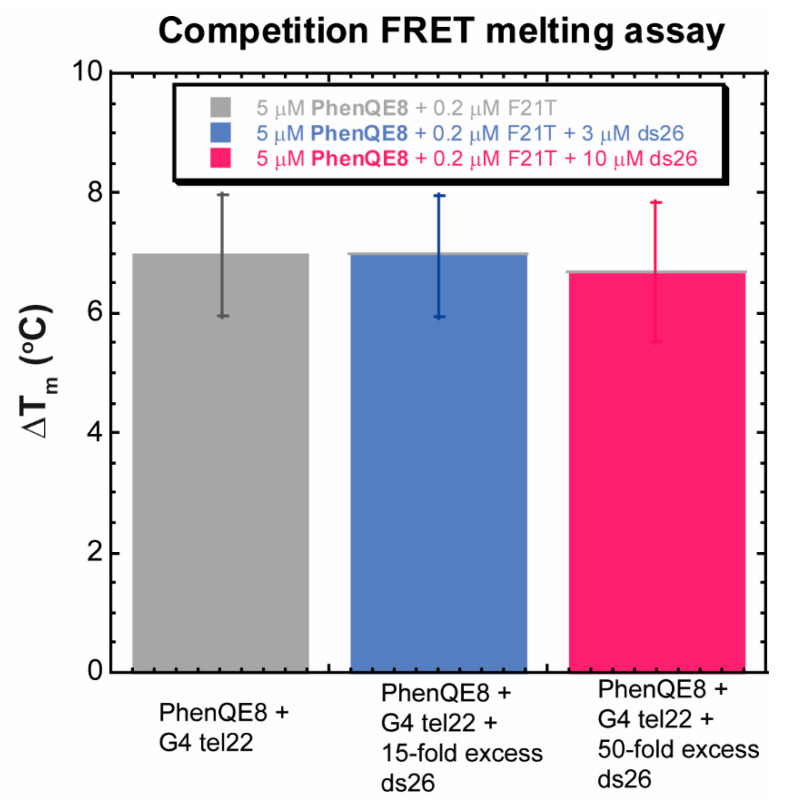
Competition DNA FRET melting experiments for PhenQE8 at a 5 μM concentration with 0.2 μM G4 sequence Tel22 (10 mM lithium cacodylate, 90 mM LiCl, 10 mM KCl) in the absence (grey) and presence (blue and dark pink) of competitor dsDNA sequence ds26: 3 μM, 15-fold DNA excess (blue) and 10 μM, 50-fold DNA excess (dark pink).

When the telomeric quadruplex was incubated with PhenQE8 at 5 μM, a ∆T_m_ = 7 ± 1 °C was observed (Figure 4). On the other hand, addition of excess of the ds26 sequence at 3 and 10 μM concentrations revealed only a decrease in the ability of the compound to stabilize the quadruplex in the latter case, and ∆T_m_ = 7 ± 1 °C and ∆T_m_ = 6.7 ± 1.1 °C were determined, respectively. These represent high selectivity values (S = 1 and S = 0.95, respectively) and indicate that PhenQE8 can remain bound to the telomeric G-quadruplex even in the presence of high excess of duplex DNA.

#### 2.2.2. Circular Dichroism 

The capability of PhenQE8 to affect the hybrid telomeric quadruplex structure was qualitatively studied by circular dichroism (CD). CD experiments cannot provide a precise structure of folded quadruplexes and their ligand complexes, but they are commonly used to assess the characteristic folding topologies of G-quadruplexes as a function of the experimental conditions (e.g., metal ions, concentration, temperature, etc.) and/or upon ligand binding [56,57,58]. Thus, CD spectra on DNA quadruplexes and their ligand complexes can reveal different binding events such as thermal stabilization, structural conformational changes induced by the ligands under investigation and possible binding sites [57]; information that can be confirmed with the use of high-resolution spectroscopic techniques (NMR, X-ray diffraction).

Circular dichroism can be thus used to distinguish between the parallel-stranded (Group I) topology from all other antiparallel quadruplexes (Group II and III topologies) [59]. In this work we exclusively focused on the human telomeric sequence Tel22, which was folded under potassium-rich ionic conditions, representing an example of the commonly known Group II hybrid (or 3 + 1) structures. The telomeric quadruplex in the presence of potassium is, in reality, a mixture of topologies. Its characteristic features are a peak at ~295 nm and a shoulder at ~270 nm, along with a trough at ~235 nm, which is indicative of the presence of mixed antiparallel-parallel structures (or two distinctive DNA hybrid topologies) [60,61]. 

**Figure 5:**
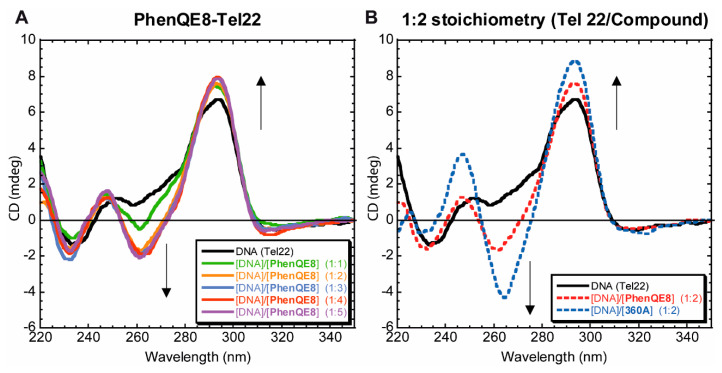
(**A**) Changes in circular dichroism (CD) spectra of quadruplex Tel22 (4 μM strand molarity), folded in the presence of potassium ions (10 mM lithium cacodylate, 90 mM LiCl, 10 mM KCl, pH 7.3, 25 °C) upon addition of PhenQE8 in a molar ratio quadruplex DNA/PhenQE8 ranging from 1:1 to 1:5. (**B**) CD changes at 1:2 Tel22/ligand stoichiometry for PhenQE8 and reference compound 360A.

The effect of PhenQE8 on the quadruplex structure was assessed by recording the CD spectra of G-quadruplex DNA, folded in the presence of K^+^ ions, in the absence and in the presence of different molar ratios DNA/ligand from 1:1 to 1:5. The changes induced on DNA CD spectra upon PhenQE8 binding are shown in Figure 5A. The intensity of the peak at 295 nm was increased as a function of an increase of compound concentration up to the 1:2 stoichiometry, whereas the intensity of the band at 270 nm was decreased. This fact, along with the appearance of a negative band at ~263 nm, revealed that PhenQE8 is a modest stabilizing agent of the quadruplex structure and, most important, the compound is able to induce a conformational change in the quadruplex structure, with a slight progressive shift from the type of structures classified as Group II (or cluster 2) towards what it resembles to fall within Group III or cluster 3 structures, with an increased antiparallel character [58]. This behavior has been observed for other known quadruplex binding ligands [62]. For comparison purposes, the CD spectrum obtained in parallel experiments with compound 360A, a reference quadruplex ligand, is also shown in Figure 5B.

A similar, but more subtle stabilizing effect was observed when ionic conditions were changed, and an increased potassium salt concentration (110 mM) was employed to simulate more physiologically relevant conditions (Figure S14). Furthermore, in both ionic conditions tested, additional analysis of the CD spectra in the 350–550 nm region (Figures S14 and S15) did not reveal the raising of a band induced by the presence of the ligand (iCD band), which is suggestive of the absence of strong groove binding or intercalative interactions with the quadruplex, and points towards end-stacking interactions [63,64].

Finally, a preliminary experiment was carried out to determine the presence or not of intermediate unfolding states in Tel22 caused by PhenQE8 by analyzing the process in FRET buffering conditions (low K^+^ concentrations) and at a higher potassium ion concentration (110 mM). The results are summarized in Figure S16. Interestingly, PhenQE8 at a 1:2 Tel22/ligand stoichiometric ratio, did not seem to exert any additional effect during the unfolding of Tel22 despite stabilization of the structure against unfolding (Figure S16, panels A and C). However, in the presence of high K^+^ concentrations (Figure S16, panels B and D), PhenQE8 not only stabilized the quadruplex structure but it produced distortions in the spectra indicative of the presence of intermediate states. This methodology [65] might be useful in future studies to obtain relevant thermodynamic information about Tel22-PhenQE8 complexation process.

All in all, the results from the different CD experiments indicated that **PhenQE8** can bind and modestly stabilize the hybrid human telomeric quadruplex sequence Tel22, likely through relatively weak end-stacking interactions, while inducing partial conformational changes towards the antiparallel structures, similarly to other known quadruplex ligands. It is important to remark, however, that **PhenQE8** does not produce further significant stabilization of the quadruplex beyond the 1:2 Tel22/ligand stoichiometric ratio, unlike other known quadruplex ligands. In addition, preliminary variable temperature experiments revealed that the ligand is able to produce intermediate unfolding states on Tel22t physiologically relevant potassium ionic concentrations.

#### 2.2.3. Noncompetition and Competition Equilibrium Dialysis 

After determining that PhenQE8 can behave as a telomeric quadruplex DNA ligand, we were interested in determining its binding association constant and in corroborating that the compound is able to discriminate between the quadruplex and duplex structures, displaying structural selectivity towards the quadruplex as shown by the FRET melting assays. Thus, equilibrium dialysis and competition equilibrium dialysis experiments were planned by using either oligonucleotide Tel22, representing the human telomeric sequence 5′-A(GGGTTA)_3_GGG-3′ [66], the previously reported double stranded ds17 sequence [67] or a combination of both in the competition version of this experiment. In the latter case, modification of the experimental protocol developed by Chaires [68], which allows a fast screening and determination of association constants of different DNA structures binding to small-molecule ligands, was chosen. 

The results obtained for PhenQE8 using telomeric G-quadruplex DNA Tel22 and double stranded DNA ds17 are shown in Table 1. Experiments were run with ~2 μM solutions of PhenQE8 equilibrated with 75 μM of nucleic acid for 24 h in a buffer containing potassium ions. At the end of the equilibration period, Triton X-100 was added to the dialysis solutions and UV-visible spectra were recorded in order to determine the concentrations of free and DNA-bound ligand. The amount of the DNA-bound ligand was averaged over at least two trials. The competition dialysis data were then used to calculate the apparent association constants of compound PhenQE8 with Tel22, with ds17 or with both structures simultaneously in a competitive fashion using the equation *K*_app_ = C_b_/(C_f_)(S_total_ − C_b_) [68], where C_b_ is the amount of ligand bound, C_f_ is the free ligand concentration and S_total_ = 75 μM in monomeric units. 

Table 1 shows that compound PhenQE8 displays a differential binding affinity for the DNA structures under investigation, with apparent association constants in the order of 10^6^ M^−1^ in the case of the telomeric quadruplex Tel22, and of 10^4^ M^−1^ in the case of dsDNA. In both competition and noncompetition versions of the assay, the compound exhibited superior binding affinity for the telomeric quadruplex than for double-stranded sequence ds17, revealing a significant preference for binding the telomeric quadruplex structures. In general, apparent association constants in competition conditions were found slightly lower in value than under noncompetition conditions, although in the same order of magnitude. Regarding the binding selectivity (roughly estimated as the ratio K_app_ (Tel22)/K_app_ (ds17) under competition conditions), the compound showed a clear preference to bind the telomeric quadruplex over dsDNA (approximately 90-fold selectivity), which represents a significant selectivity. To further corroborate the relatively lower affinity towards dsDNA, the experiment was also performed with calf thymus (CT) DNA, revealing similar binding affinity towards this long DNA sequence than towards the short sequence ds17. Overall, it was found that the compound shows high binding selectivity towards quadruplex DNA Tel22 in comparison to duplex DNA. These results are in good agreement with the results obtained in the competition FRET assays.

#### 2.2.4. CT DNA Viscometric Titration 

Having established that PhenQE8 preferentially binds the telomeric quadruplex but it can bind double-stranded DNA as well, we wanted to investigate whether the compound recognizes duplex DNA either by intercalation between the DNA base pairs, by binding to the DNA grooves, or externally. It is well known that viscosity measurements provide a simple way to study the binding modes of dsDNA ligands [69]. In particular, viscometric experiments can be readily used to establish and distinguish between different noncovalent DNA binding modes, such as DNA intercalation and groove binding. According work of Cohen and Eisenberg [70], the gradual titration of DNA solutions with the compounds of interest can produce linear plots of the cubed root of the relative DNA viscosity (η/η_o_)^1/3^ versus the molar ratio of bound ligand to DNA nucleotide (r). The slope values in these plots correlate well with the DNA-ligand binding modes. Groove binding compounds normally display a slope close to 0.0, whereas classical monointercalants result in a slope close to 1.0 [69,70]. Experimentally, the slopes associated with minor-groove binder compounds range from −0.3 to 0.2 [71].

**Figure 6:**
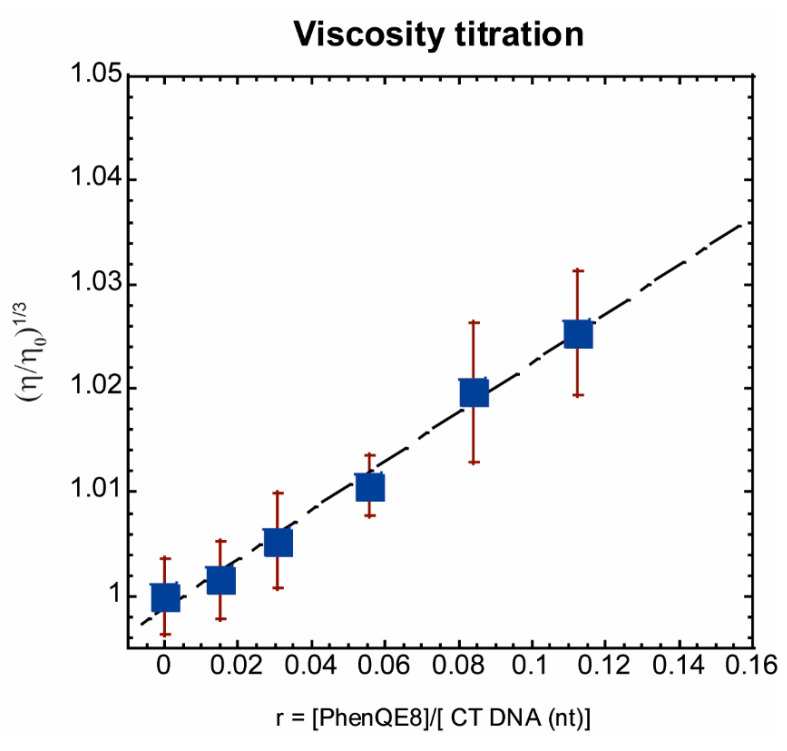
Viscometric titration experiment of calf thymus (CT) DNA and ligand PhenQE8 at 25 °C (10 mM sodium phosphate buffer, pH 7.2). Averaged slope value over replicate experiments is 0.24 ± 0.12.

Viscometric measurements were carried out at 25 ± 0.01 °C by adding small aliquots of compound PhenQE8 to the DNA solutions of calf thymus (CT) DNA, in 10 mM sodium phosphate buffer (pH 7.2). Flow times were recorded in the presence (η) and in the absence (η_o_) of compound. The viscosity data were plotted as (η/η_o_)^1/3^ versus r, as shown in Figure 6.

Compound PhenQE8 demonstrated a linear (η/η_o_)^1/3^ versus r correlation in the typical r range used in these experiments, with a higher dispersion of experimental data at higher ligand/DNA ratios, which might indicate a slight change in the linear trend caused by the implication of two different binding modes depending on the concentration of the ligand. Overall, the mean slope value obtained from the viscosity plots from replicate experiments in the range of concentration tested was found to be 0.24 ± 0.12. These results confirm that PhenQE8 does not interact with double stranded DNA primarily by an intercalation mechanism, but preferentially by groove binding, external binding or partial intercalation. Structurally related to PhenQE8, there are several examples in the literature of guanidinium-based DNA ligands that bind the double helix through the minor groove, such as DB950 [72], and certain fluorene [73], diaryl [74,75] and triaryl derivatives [76], among others. Although not directly extrapolatable, due to the differential number and dimensions of the grooves and to the different dynamics of the two secondary DNA structures, it is feasible that when PhenQE8 interacts with the human telomeric quadruplex the recognition of the grooves and/or loops of the structure may have a contribution, besides π-π stacking interactions, which have been found not significant in its binding to duplex DNA.

#### 2.2.5. Antitumor Activity in Cultured Cells

Finally, the preliminary antitumor activity of PhenQE8 in cultured tumor cells PC-3 (prostate), HeLa (cervix), and MCF-7 (breast) was studied by the MTT 3-(4,5-dimethylthiazol-2-yl)-2,5-diphenyltetrazolium bromide (MTT) assay. For comparison purposes, healthy fibroblast (HFF-1) and healthy prostate (RPWE-1) cell lines were also included in this study as controls for cytotoxicity in nontumor cells. The selective quadruplex ligand 360A, as well as chemotherapeutic drug cisplatin, were additionally tested to allow a preliminary assessment of the antitumor potency of PhenQE8 when compared to these known cytotoxic agents. 

The results from this assay are shown in Table 2. These experiments revealed that PhenQE8, after a 72 h treatment, is cytotoxic in the low micromolar concentration range in the case of PC-3 and HeLa cells (IC_50_ values ~6 and 16 μM, respectively), whereas the antitumor activity of the compound is considerably lower in MCF-7 cells (~53 μM). These values represent a higher cytotoxicity than cisplatin (IC_50_ values ~ 13, 18 and 97 μM, respectively) although lower than G-quadruplex ligand 360A (IC_50_ values ~ 3, 9 and 45 μM, respectively) (Table 2). Most importantly, the results with the HFF-1 cell line showed that the compound toxicity in healthy human fibroblasts under the experimental conditions tested is very low when compared to the reference cytotoxic compounds 360A and cisplatin, with a IC_50_ ~170 μM, which represents at least a five-fold reduced cytotoxicity respect to the reference cytotoxic agents. The selectivity indexes (SI) of PhenQE8, estimated as the ratios between toxicities in healthy cells and in tumor cells were found to be ~ 30, 10 and 3, for PC-3, HeLa and MCF-7, respectively. Further testing was then carried out with a second healthy cell line. In this case the normal prostate cell line RPWE-1 produced a similar trend, with PhenQE8 displaying lower cytotoxicity than the reference cytotoxic agents, although the effect observed was not as pronounced as with HFF-1 (~4-fold and 2-fold reduced cytotoxicity in comparison to 360A and cisplatin, respectively).

Taken together, the results from these initial MTT assays reveal that the new compound PhenQE8 shows promising biological activity based on its antitumor potency and in its potential selectivity against malignant cells. Therefore, further experiments should be performed to corroborate these findings and to gain a deeper insight into the mechanisms of action that are responsible for the interesting biological properties observed. 

## 3. Conclusions

In summary, we have reported herein the preparation of a novel telomeric G-quadruplex ligand based on 1,10-phenanthroline and containing two guanyl hydrazone functions in the side chains, which are expected to be protonated under physiological conditions. The designed ligand, PhenQE8, is a polynitrogenated compound that, besides the 1,10-phenanthroline heterocyclic core, lacks the additional aromatic or heteroaromatic rings that are commonly found in other known G-quadruplex stabilizers and contribute with their surface to maximize the π-π stacking interactions that characterize their recognition of the terminal tetrads of G-quadruplexes. Despite the limitation of a scarce aromatic planar surface, the presence of the guanyl hydrazone groups may favor its interactions with the quadruplex, e.g., by hydrogen bonding with the grooves and/or loops of the telomeric quadruplex. Moreover, X-ray diffraction studies revealed that the molecule can adopt an extended conformation, with one molecular dimension of ~16 Å, which may be optimal for quadruplex recognition and for partial discrimination of quadruplex versus duplex structures.

As the target G-quadruplex DNA sequence, we focused on the human telomeric sequence, which is one of the most representative examples of polymorphic quadruplex structure. In the present study we investigated the hybrid or mixed quadruplex structures, which represent a mixture of topologies and are formed under potassium-rich conditions. This mixture of topologies is thought to be more relevant, and closer to the physiological conditions, than, for example, the structure formed in the presence of sodium ions. Under these conditions, we studied the in vitro interactions of PhenQE8 through several techniques: fluorescence-based assays (DNA FRET melting experiments, both noncompetitive and competitive), circular dichroism and equilibrium dialysis. In addition, interaction of the ligand with double stranded CT DNA was studied by viscosity titrations in an attempt to gain an insight into the interaction mode with the duplex structure, to which PhenQE8 binds as well, although with a significantly lower affinity than to the quadruplex.

The interaction studies revealed that the compound can thermally stabilize the telomeric quadruplex DNA, whereas it exerts an insignificant effect on double stranded DNA. Competition FRET melting assays suggested that the compound maintains its ability to bind the telomeric quadruplex even with the addition of a significant stoichiometric excess of duplex DNA, therefore displaying high binding selectivity. From a structural point of view, the CD titration experiments showed that the compound can modestly stabilize the hybrid structures of the telomeric DNA in different potassium ion concentrations, with minimal stabilization beyond the 1:2 Tel22/ligand ratio and, most important, it seems to induce minor conformational changes, with a slight shift from the hybrid structure to a structure, with an increased antiparallel character. This behavior is not exclusive to the novel ligand PhenQE8, but it has also been reported for other standard quadruplex ligands [62]. Furthermore, the absence of a CD signature, specifically of an iCD band in the 350–550 nm region of the spectra, suggested weak groove binding or intercalative interactions with the quadruplex. PhenQE8, however, might be able to induce the presence of intermediate unfolding states in Tel22 under high potassium salt conditions, and a future detailed study of this process could be used to gain important thermodynamic information about its binding to telomeric DNA. 

In addition, PhenQE8 binds to quadruplex and double-stranded DNA with different affinities. Equilibrium dialysis experiments revealed apparent association constants in the order of 10^6^ M^−1^, whereas the compound binds to duplex DNA with an affinity close to two orders of magnitude lower. When the experiment was carried out in competition conditions, a selectivity in the binding of PhenQE8 to the telomeric quadruplex versus to the duplex DNA ds17 was estimated to be ~90-fold. On the other hand, viscosity assays on CT double stranded DNA revealed that the compound does not intercalate into DNA base pairs, but it likely binds the double helix by groove or external binding, similar to other DNA ligands containing guanidinium groups. Taken together, the results described in this article seem to point to an interaction mode that stabilizes modestly the telomeric quadruplex and that might involve, at least in part, a relatively weak recognition of the DNA grooves and/or loops besides the classical, π-π stacking interactions with the terminal tetrads of the G-quadruplex.

Finally, the antitumor potency of the ligand and toxicity were preliminary studied in cultured cells by using the MTT assay. The compound showed interesting biological activity in the panel of cells tested (PC-3, HeLa and MCF-7) with IC_50_ values in the micromolar concentration range, revealing lower potencies than the G-quadruplex ligand 360A, but higher than chemotherapeutic drug cisplatin. Most significantly, the compound cytotoxicity in healthy cells, such as HFF-1 and RWPE-1, is considerably diminished in comparison to the cytotoxic reference agents, therefore providing better selectivity indexes. 

Based on these results, we envision that PhenQE8 might constitute a good starting point for the development of other telomeric (and perhaps nontelomeric) quadruplex binding analogues with increased activity and higher selectivity indexes. Further investigation on the mechanism of action is needed, including an in-depth evaluation of biological activity. Optimization of pharmacokinetic properties by using, for example, specific nanocarriers, is currently under way.

## 4. Materials and Methods

### 4.1. General Methods

All reagents were commercially available (Sigma-Aldrich, Madrid, Spain; Janssen Cilag, Madrid, Spain) in high purity and used as received. Solvents were purchased from commercial suppliers and were used without further purification. Analytical Thin layer chromatography (TLC) was performed on precoated aluminum silica gel sheets (Merck 60 F_254_ 0.25 mm) and the chromatograms were visualized using UV radiation at 254 or 366 nm. Melting points were determined on an Electrothermal Digital IA9100 apparatus and were uncorrected. IR spectra were recorded on an IR FT PerkinElmer (Spectrum 2000) spectrophotometer using KBr disks or NaCl windows. ^1^H NMR and ^13^C NMR spectra were recorded on a Bruker 400 Ultrashield in DMSO-d_6_ or D_2_O (or DMSO-d_6_ with 2.5% D_2_O) at 298 K using standard pulse sequences. Chemical shifts were reported relative to the residual DMSO-d_6_ (*δ*_H_ 2.50, 3.33 ppm), DMSO-d_6_ (*δ*_C_ 39.5 ppm) or, in the case of D_2_O, to an internal acetone standard (*δ*_H_ 2.22 ppm) [77]. Coupling constants (J values) were given in Hz. Resonance patterns were designated with the notations s (singlet), d (doublet), m (multiplet), br s (broad singlet). Electrospray ionization mass spectra (ESI-MS) were recorded using a Thermo Scientific TSQ Quantum Triple Quadrupole LC-MS or a MAXIS II (SiDI, Universidad Autónoma de Madrid, Madrid, Spain). 

For DNA interaction studies, distilled, deionized water (ddH_2_O, Milli-Q) was used for the preparation of all buffers and aqueous solutions. The reagents and solvents molecular biology grade and were utilized as provided by Sigma-Aldrich. Oligonucleotides were acquired HPLC-purified and desalted from IDT^®^ (Integrated DNA Technologies, Leuven, Belgium). DNA samples were dissolved in BPC grade water.

UV-visible spectra were recorded using a Perkin-Elmer Lambda 35 spectrophotometer and CD spectra were run on a Jasco J-715 spectropolarimeter. FRET DNA melting assays were performed on an ABI PRISM^®^ 7000 Sequence Detection System (Applied Biosystems, Madrid, Spain). Stock solutions of compound PhenQE8 at millimolar concentrations were made freshly before each experiment in pure BPC grade water and 10% DMSO and subsequently diluted to micromolar concentrations in adequate buffer or cell culture media. MTT assays were carried out at the Cell Culture Unit in UAH following standard protocols and using an ELISA plate reader (ELX 800 Biotek Instruments, Madrid, Spain).

### 4.2. Synthesis and Structural Characterization

1,10-phenanthroline-2,9-carbaldehyde. Over a stirring solution of neocuproine (2,9-dimethyl-1,10-phenanthroline, 300 mg, 1.44 mmol) in 20 mL of dioxane/H_2_O (96:4, *v*/*v*) SeO_2_ (720 mg, 6.49 mmol) was added. The reaction was stirred at reflux for 3 h. Afterwards, the mixture was filtered over celite, washed with hot dioxane and the filtrate was evaporated. Then, the crude was dissolved with EtOH, precipitated with cold Et_2_O and filtered to obtain 1,10-phenanthroline-2,9-carbaldehyde (306 mg, 90%) as a beige solid. M.p. [52]: 243–244 °C. IR (KBr), *ν*_max_ (cm^−1^) [52]: 3600–2800, 1625, 1515, 1120, 1105, 1005, 1000, 875, 825. ^1^H-NMR (400 MHz, DMSO-d_6_), δ (ppm): 10.34 (s, 2H, CHO), 8.77 (d, J = 8.2 Hz, 2H, H-4,7), 8.29 (d, J = 8.2 Hz, 2H, H-3,8), 8.26 (s, 2H, H-5,6). ^13^C-NMR (75 MHz, DMSO-d_6_), δ (ppm) [52]: 194.1 (CHO), 152.6 (C-2,9), 145.7 (C-10a,10b), 138.9 (C-4,7), 131.9 (C4a,6a), 129.7 (C-5,6), 120.6 (C-3,8). MS (ESI, *m*/*z*) for C_14_H_9_N_2_O_2_ [M + H]^+^ [52]: calculated: 237.07; found: 237.12.

(2E,2′E)-2,2′-((1,10-phenanthroline-2,9-diyl)bis(methanylylidene))bis(hydrazine-1-carboximidamide). Over a stirring solution of 1,10-phenanthroline-2,9-carbaldehyde (30 mg, 0.13 mmol) in aqueous 37% HCl (0.5 mL) a solution of aminoguanidine bicarbonate (36.2 mg, 0.26 mmol) in H_2_O (1.0 mL) was added dropwise. The reaction mixture was then heated at reflux for 1 h, cooled at r.t. and KOH (0.6 mL 40% (*v/v*)) was added. The mixture was heated at reflux during 10 min and the solvent was removed by filtration. The resulting solid was treated with MeOH, and salts were filtered off. This procedure was repeated twice. The desired product dissolved in a minimal amount of MeOH and precipitated with cool Et_2_O, filtered and washed thoroughly with cool Et_2_O to provide compound PhenQE8 (50.4 mg, 68%), as a yellow solid.

M.p.: >200 °C (decompose). IR (NaCl) *ν*_max_ (cm^−1^): 3435, 2998, 2914, 1992, 1663, 1437, 1407, 1312, 1027, 954, 933, 898, 703, 670. ^1^H NMR (400 MHz, D_2_O) δ (ppm): 8.00 (s, 2H), 7.86 (d, J = 8.0 Hz, 2H), 7.65 (d, J = 8.0 Hz, 2H), 7.43 (s, 2H). ^1^H NMR (400 MHz, DMSO-d_6_, 0.025M) δ (ppm): 8.72 (d, J = 8.0 Hz, 2H, H-3,8), 8.63 (d + s overlapped, J = 8.0 Hz, 4H, H-4,7, CH=N), 8.10 (s, 2H, H-5,6), ~8.15–7.90 (br s, 8H, NH). ^1^H NMR (400 MHz, DMSO-d_6_ + 2.5% D_2_O, 0.025 M) δ (ppm): 8.68 (d, J = 8.0 Hz, 2H, H-3,8), 8.61 (d + s overlapped, J = 8.0 Hz, 4H, H-4,7, CH=N), 8.09 (s, 2H, H-5,6). ^13^C NMR (101 MHz, DMSO-d_6_+ 2.5% D_2_O, 0.025 M) δ (ppm): 155.4 (C_guan_), 152.6 (C-2,9), 147.5 (CH=N), 144.4 (C-10a,10b), 137.9 (C-4,7), 129.5 (C-4a,6a), 127.8 (C-5,6), 121.0 (C-3,8). HRMS-ESI *m*/*z* [M + H]^+^: calculated for C_16_H_17_N_10_: 349.1632, found: 349.1622.

X-ray structure of PhenQE8. After elimination of the solvent from the reaction mixture, the resultant solid was dissolved in methanol. The addition of diethyl ether led to a white precipitate. Colorless crystals of PhenQE8 were grown by slow evaporation at room temperature from a dimethyl sulfoxide solution of the white precipitate. The crystals were removed from the vial and covered with a layer of a viscous perfluoropolyether (FomblinY). Suitable crystals, selected with the aid of a microscope, were mounted on a cryoloop and placed in the low temperature nitrogen stream of the diffractometer. The intensity data sets were collected at 200 K on a Bruker-Nonius KappaCCD diffractometer equipped with an Oxford Cryostream 700 unit. Crystallographic data for PhenQE8 are presented in Table S1. 

The structure was solved, using the WINGX package [78], by intrinsic phasing methods (SHELXT) [79], and refined by least-squares against *F*^2^ (SHELXL-2014/7) [80]. Compound PhenQE8 crystallized with solvent molecules (water, dimethyl sulfoxide) and/or salts (KCl, K_2_CO_3_), which were found in the difference Fourier map, but it was not possible to obtain a chemically sensible model for them, so the Squeeze [81] procedure was used to remove their contribution to the structural factors. The asymmetric unit was formed by one and a half molecules of PhenQE8 (Figure S12) associated by π-π interactions. All nonhydrogen atoms were anisotropically refined, whereas all the hydrogen atoms were positioned geometrically and refined by using a riding model. 

### 4.3. DNA Interactions and Antitumor Activity

#### 4.3.1. Fret DNA Melting Assays

Labeled oligonucleotides F21T (5′-FAM-(GGG TTA)_3_GGG-TAMRA-3′) and F10T (5′-FAM-TAT AGC TAT A/Sp18/TAT AGC TAT A-TAMRA-3′) were dissolved in BPC grade water to provide 50 μM stock solutions. 0.25 μM solutions of F21T and F10T were then prepared by mixing the DNA stock solution (50 μM), the corresponding 2X buffer (K^+^ conditions) and water. These solutions were heated at 90 °C for 5 min and then cooled at 0 °C (F21T) or gradually to room temperature (F10T) for three hours. The solutions were allowed to stand overnight at 4 °C. 

Compound PhenQE8 was dissolved in water and a maximum of 0.5% DMSO (50 μM stock solution) and a concentration range from 0 to 10 μM was assayed using the oligonucleotide sequences F21T and F10T. Each well of the 96-well microplate contained a 50 μL total volume with a 200 nM oligonucleotide concentration in the corresponding buffer pH = 7.3. F21T (K^+^) contained 10 mM potassium chloride, 90 mM lithium chloride and 10 mM lithium cacodylate; F10T contained 100 mM lithium chloride and 10 mM lithium cacodylate. 

The experiments were performed on an ABI PRISM^®^ 7000 Sequence Detection System (Applied Biosystems). The melting procedure included a 5-min incubation at 24 °C followed by a temperature ramp at a 1°C/min rate with fluorescence measurement at every degree up to 95 °C and subsequently a 5-min incubation at 96 °C followed by a temperature ramp at a −1 °C/min rate with fluorescence measurement at every degree down to 25 °C. The melting curves were obtained as a change in emission of FAM (6-carboxyfluorescein) excited at 492 nm and emitting at 516 nm. Experiments were run at least in duplicate. The melting temperatures (T_m_) were determined from normalized curves as the mid-transition T_1/2_ temperatures. T_1/2_ represents an apparent melting temperature, defined as the temperature where the normalized fluorescence has a value of 0.5.

The competition FRET melting assays were carried out by employing analogous experimental conditions and following the same protocol as the noncompetition experiments, with F21T and a compound concentration of 5 μM, in the absence or in the presence of DNA duplex competitor sequence ds26 (5′-CAA TCG GAT CGA ATT CGA TCC GAT TG-3′). Experiments were run in triplicate. Two different dsDNA concentrations were tested: 3 μM (15-fold duplex excess) and 10 μM (50-fold duplex excess). Selectivity of quadruplex versus duplex binding (S) was calculated at the two dsDNA tested concentrations according to the formula S = (∆T_m_)_competitor_/(∆T_m_)_w/o competitor_.

#### 4.3.2. Circular Dichroism Spectra

The CD spectra were obtained using a Jasco J-715 spectropolarimeter. CD experiments were carried out with the DNA oligonucleotide sequence 5′-A(GGGTTA)_3_GGG-3′ (Tel22, Table S2) folded into G-quadruplex at the concentration of 4 μM or 8 μM (strand molarity) in analogous experimental conditions to those used in DNA FRET melting assays. The buffer system contained a 110 mM total salt concentration—10 mM lithium cacodylate, 90 mM LiCl, 10 mM KCl at pH 7.3. In addition, parallel experiments were run using 10 mM potassium phosphate buffer and 110 mM KCl. Solutions of the DNA and ligand PhenQE8 at DNA/ligand ratios varying from 1:1 to 1:5 were prepared two hours prior measurement. The CD spectra were recorded at 25 °C using a 0.5 cm-path cell, 1 nm band width and 0.5 nm intervals. The CD spectra were averaged over two scans. 

For the preliminary analysis of intermediate unfolding states, Tel22 (4 μM) was heated from 30 to 90 °C, at 5 °C intervals in the absence and presence of PhenQE8 at a DNA/ligand ratio of 1:2, employing analogous experimental conditions (buffers and equipment settings) as the assays at 25 °C.

#### 4.3.3. Equilibrium Dialysis 

Several dialysis assays were performed with quadruplex DNA and/or with dsDNA. The unlabeled G-quadruplex DNA (Tel22 sequence) and dsDNA (ds17 sequence) were purchased from IDT^®^, HPLC-purified and desalted. Tel22 is 5′-A(GGGTTA)_3_GGG-3′; ds17 is the pair 5′-GGG TTA CTA CGA ACT GG-3′/5′-CCA GTT CGT AGT AAC CC-3′. Duplex DNA from calf thymus (CT DNA), (deoxyribonucleic acid, activated, type XV), was directly purchased from Sigma Aldrich and used as provided. A 10 mM potassium phosphate buffer (pH = 7.2) with a KCl salt concentration of 100 mM was employed in the preparation of all nucleic acid stock solutions. The concentrations of the nucleic acid solutions were determined by UV-visible at 90 °C spectrophotometry using the λ_max_ values and extinction coefficients provided by the manufacturer and listed in Table S2 (Supplementary Materials). Extinction coefficients of PhenQE8 were determined in water and in buffer in the absence and in the presence of 1% (*w*/*v*) surfactant (Table S3, Supplementary Materials). Competition and noncompetition dialysis experiments were performed following a similar protocol as the one described by Chaires [68]. For each dialysis assay, a 0.2 mL volume of each G-quadruplex or duplex DNA in buffer (75 μM in monomeric units, tetrads or base pairs) was pipetted into individual dialysis units (Biotech Regenerated Cellulose (RC) membrane, part number 133192, Spectrum Laboratories, Inc., Breda, The Netherlands). The dialysis units were then placed in a beaker containing 200 mL of a 2 μM solution of compound PhenQE8 in buffer. The beaker was covered with parafilm and wrapped in aluminum foil, and its contents were allowed to equilibrate with continuous stirring for 24 h at room temperature (22 °C). At the end of the equilibration period, the DNA solutions inside the dialysis units were carefully transferred into microcentrifuge tubes and a 10.0% (*w*/*v*) stock solution of surfactant was added to give a final concentration of 1.0% (*w*/*v*). These solutions were allowed to equilibrate for 2 h, after which the total concentration of the ligand (C_t_) was determined by UV-visible absorbance measurements using the determined extinction coefficient for free ligands in the presence of 1.0% surfactant. An appropriate correction for the slight dilution of the sample resulting from the addition of the surfactant stock solution was made. The concentration of free compound PhenQE8 (C_f_) was also determined spectrophotometrically using an aliquot of their dialysate solution. The amount of DNA-bound compound (C_b_) was then calculated by the difference C_b_ = C_t_ − C_f_ and apparent association constants (K_app_) determined.

#### 4.3.4. Viscometric Titration 

The viscometric measurements were performed in a Visco System AVS 470 at 25 °C, using a microUbbelohde (K = 0.01) capillary viscometer. Solutions of DNA (calf thymus, CT) and compound PhenQE8 were prepared in sodium phosphate buffer (10 mM, pH = 7.2). DNA solutions (0.3–0.4 mM, in nucleotides) were equilibrated for 20 min at 25 °C and then 20 flow times were registered. Small aliquots (ca. 30–60 μL) of solutions of compound PhenQE8 (1 mM) were added next. Before each flow time registration, the solutions were equilibrated for at least 20 min at 25 °C and then 20 flow times were measured. With the averaged flow times and the viscometer constant, the viscosities (η) for each point were calculated, with η_o_ representing the DNA solution viscosity in the absence of compound. The viscosity results were then plotted as (η/η_o_)^1/3^ versus the molar ratio of bound ligand to DNA nt (r). The experiment was performed twice.

#### 4.3.5. Cell Culture and MTT Colorimetric Assay

Human PC-3 (prostate), HeLa (cervix) and MCF7 (breast) tumor cells, and healthy HFF-1 (fibroblast) and RPWE-1 (prostate) cells, were purchased from American Type Culture Collection (ATCC). Cells and culture media were: PC-3 (CRL-1435^TM^, RPMI), HeLa (CCL-2^TM^, DMEM), MCF-7 (HTB-22^TM^, DMEM), HFF-1 (SCRC-1041^TM^, DMEM), RPWE-1 (CRL-11609^TM^, Keratinocyte-SFM). Culture media were acquired from Sigma: RPMI 1640, DMEM + 10% FBS (Fetal Bovine Serum) + 10% antibiotic (penicillin/streptomycin/amphotericin B); in the case of RWPE-1 cells, media and supplements were acquired from ThermoFisher (17005075). MCF7 cells were, in addition, supplemented with insulin (Sigma, Ref. I1882, 0.001 mg/mL in 500 mL media). Cells were maintained at 37 °C in the presence of 5% CO_2_, renewing culture media three times per week. On the seeding day, when cells reached 80–100% confluence, cells were trypsinized, centrifuged and the concentration adjusted to the required experiment concentration. Cells were then seeded at a density of 10,000 cells/well into 24-well plates and treated with different concentrations of ligand PhenQE8 or the cytotoxic agents cisplatin and 360A (typically to cover the 10 nM–200 µM range), from freshly prepared stock solutions in culture media. The experiments were run in triplicate, in a total 0.55 mL well volume. After 72 h incubation (37 °C/5% CO_2_), 50 μL of MTT (5 mg/mL) was added followed by incubation for another 4 h. Then, the medium in each well was replaced by DMSO (0.5 mL) and the absorbance in every well assessed at 570 nm in an ELISA plate reader (ELX 800 Biotech Instruments, Spain). Absorbance values were normalized against negative controls (untreated cells, in triplicate) and the percentage of viable cells versus compound logarithmic concentration was plotted. IC_50_ values were determined through the equation 1/(1 + 10 ^(m2 * (log(m1) − x))^); m1 = 0.000003; m2 = 1 using Kaleidagragh^TM^ (3.52) software (Reading, PA, USA).

## Figures and Tables

**Table 1:** Apparent association constants of PhenQE8 with quadruplex DNA Tel22 and with ds DNA (ds17 and CT DNA) obtained by noncompetition and competition equilibrium dialysis in ionic potassium conditions (10 mM potassium phosphate buffer, 100 mM KCl, pH = 7.2).

	PhenQE8 + Tel22	PhenQE8 + ds17	PhenQE8 + Tel22 + ds17	PhenQE8 + CT
DNA	*K*_app_(M^−1^) × 10^−6^	*K*_app_(M^−1^) × 10^−6^	*K*_app_(M^−1^) × 10^−6^	*K*_app_(M^−1^) × 10^−6^
Tel22 (Q)	2.3 ± 0.5	--	1.35 ± 0.26	--
ds17 (D)	--	0.029 ± 0.004	0.015 ± 0.005	--
CT dsDNA	--	--	--	0.058 ± 0.013

**Table 2:** Cytotoxic activity of PhenQE8 and cisplatin in tumor PC-3 (prostate), HeLa (cervix), and MCF-7 (breast) cultured cells and in healthy HFF-1 (fibroblasts) and RWPE-1 (prostate) cells, after a 72-h incubation period.

	PC-3	HeLa	MCF-7	HFF-1	RWPE-1
Compound	IC_50_ (μM)	IC_50_ (μM)	IC_50_ (μM)	IC_50_ (μM)	IC_50_ (μM)
PhenQE8	5.6 ± 0.3	16.2 ± 1.6	52.7 ± 1.0	170 ± 23	38.8 ± 0.5
360A	2.5 ± 0.8	8.5 ± 1.4	44.9 ± 9.2	30.6 ± 2.3	9.9 ± 1.0
cisplatin	12.8 ± 0.9	18.0 ± 1.9	96.5 ± 6.7	36.6 ± 4.4	16.4 ± 1.4

## Data Availability

Data is contained within the article or supplementary material.

## Supplementary Materials

The following are available online at https://www.mdpi.com/1422-0067/22/2/749/s1.
