# Supplementary material for: PhenQE8, a Novel Ligand of the Human Telomeric Quadruplex
Source: Int J Mol Sci. 2021 Jan 13;22(2):749. doi: 10.3390/ijms22020749 (PMC7828518; doi:10.3390/ijms22020749)
Supplement: Supplementary file 1 [file ijms-22-00749-s001.zip › ijms-1039650/SupplementaryMaterials/Supporting Materials_IJMS_Gude_revised_highlighted.pdf]

Supporting Materials

PhenQE8, a novel ligand of the human telomeric quadruplex

Patricia B. Gratal, Julia G. Quero, Adrián Pérez-Redondo, Zoila Gándara,* and Lourdes Gude*

Departamento de Química Orgánica y Química Inorgánica, Instituto de Investigación Química "Andrés M. del Río" (IQAR), Universidad de Alcalá, 28805-Alcalá de Henares (Madrid), Spain

*Correspondence: zoila.gandara@uah.es (Z. G.), lourdes.gude@uah.es (L. G.)

Table of Contents

1.- Supplementary figures S1-S11: NMR and Mass Spectra Data

2.- Supplementary Table 1 and Supplementary Figure S12: x-Ray diffraction data

3.- Supplementary Figure S13. FRET assays (heating and cooling curves)

4.- Supplementary figures S14-S16: Additional CD experiments

5.- Supplementary Table 2: Oligonucleotide Sequences

6.- Supplementary Table 3: Extinction Coefficients for dialysis experiments

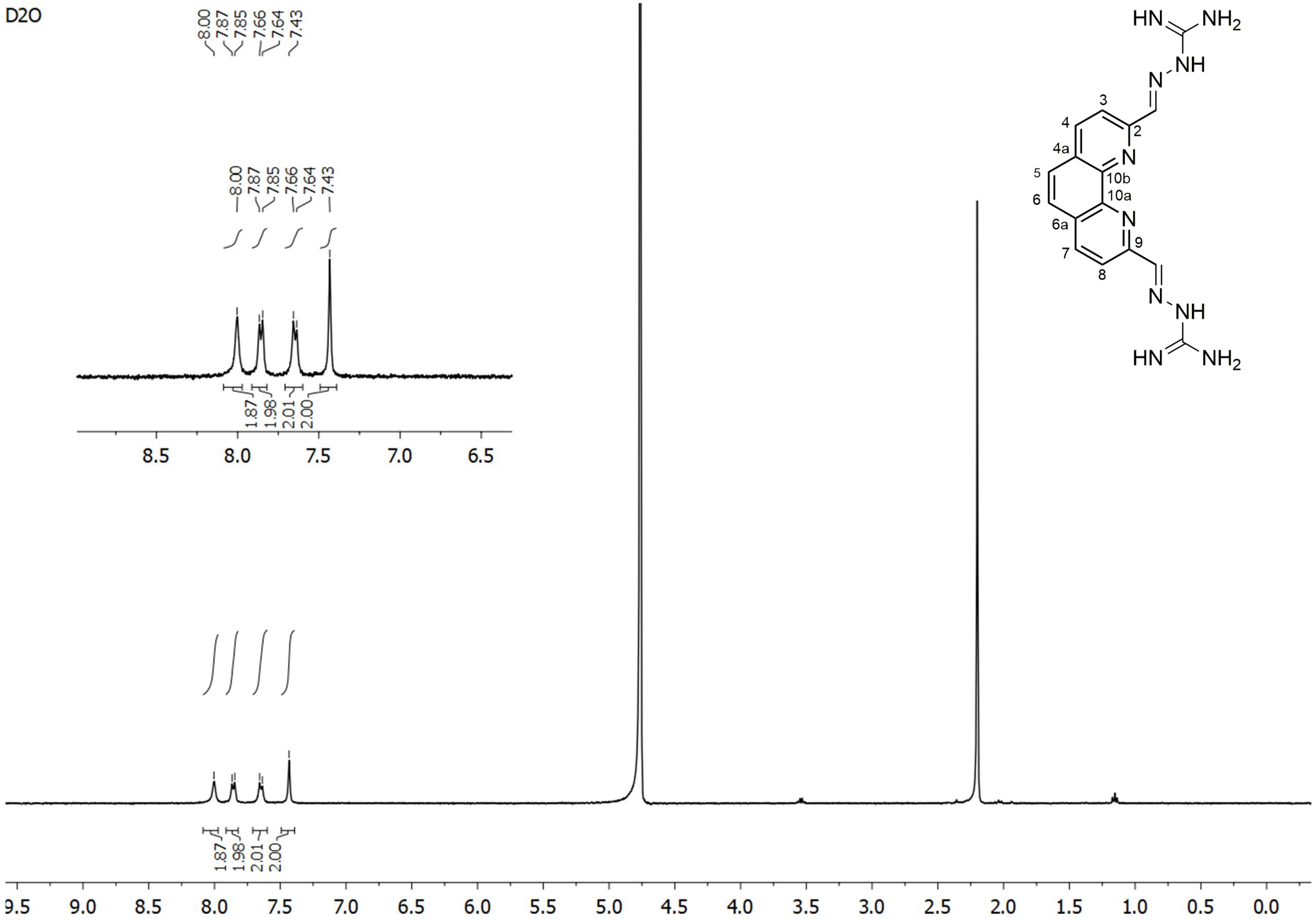

Figure S1. ^{1}H NMR (400 MHz, D_2O) of PhenQE8

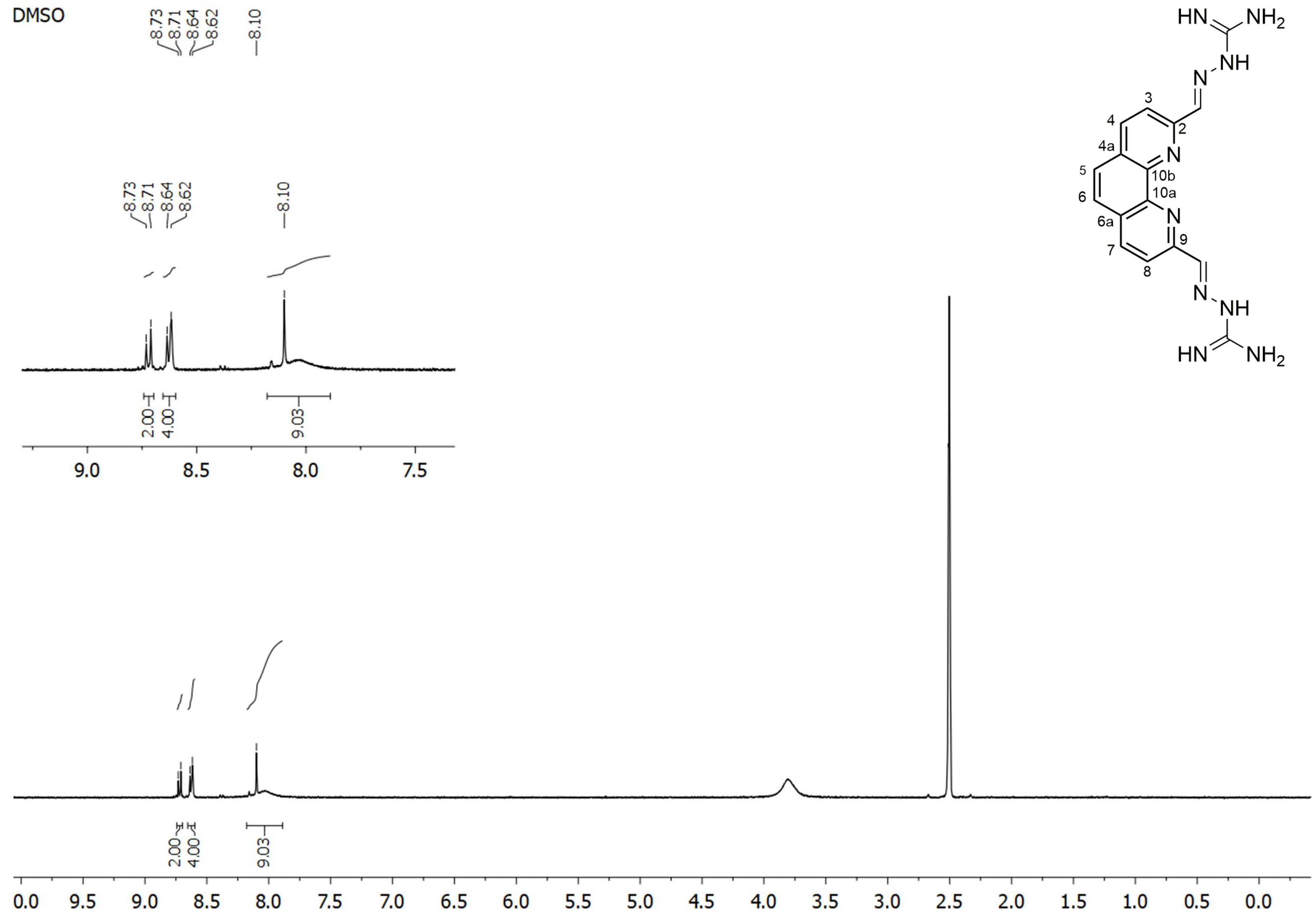

Figure S2. ^{1}H NMR (400 MHz, DMSO-d$_6$) of PhenQE8

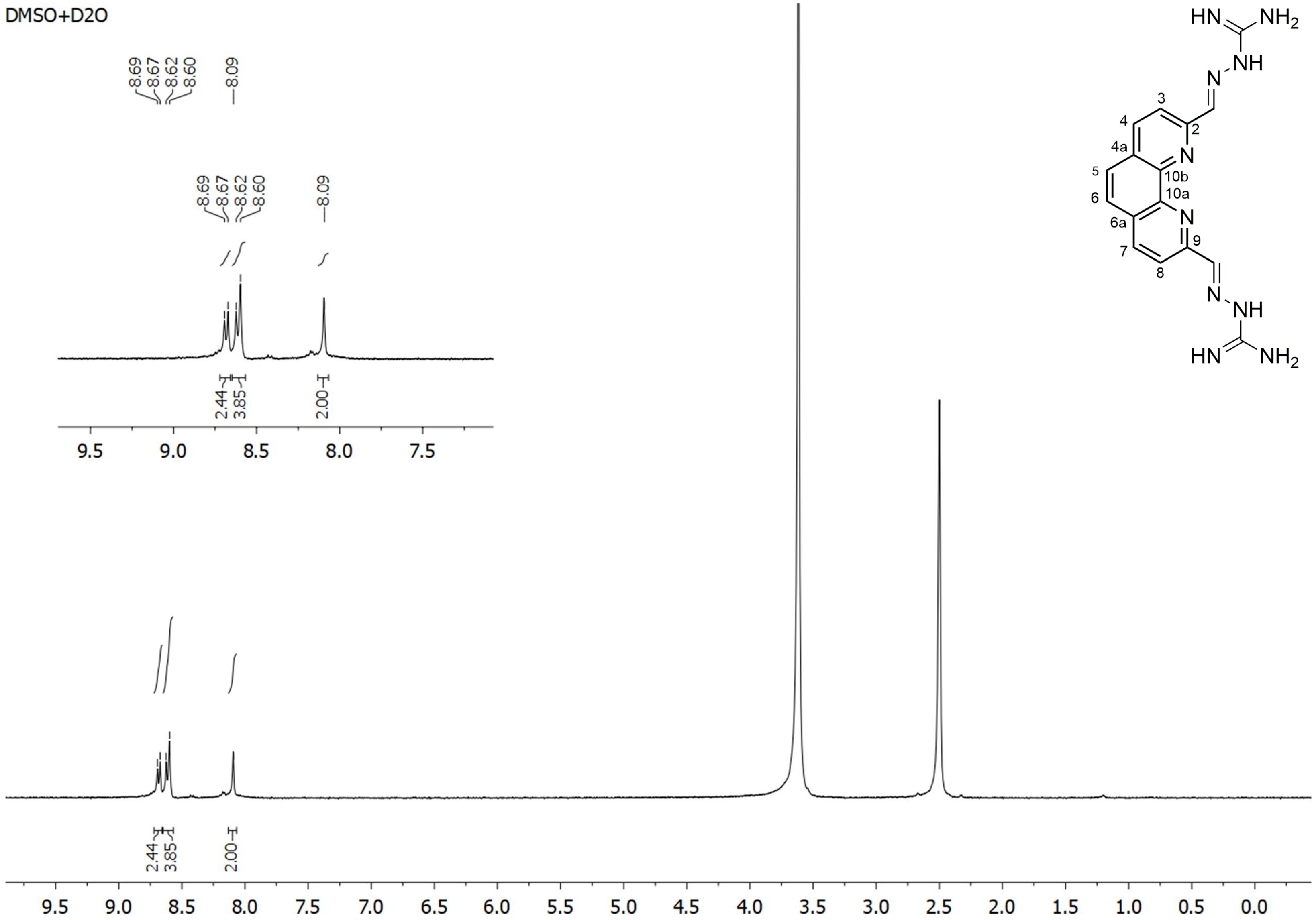

Figure S3. ^{1}H NMR (400 MHz, DMSO-d$_6$, 2.5% D$_2$O) of PhenQE8

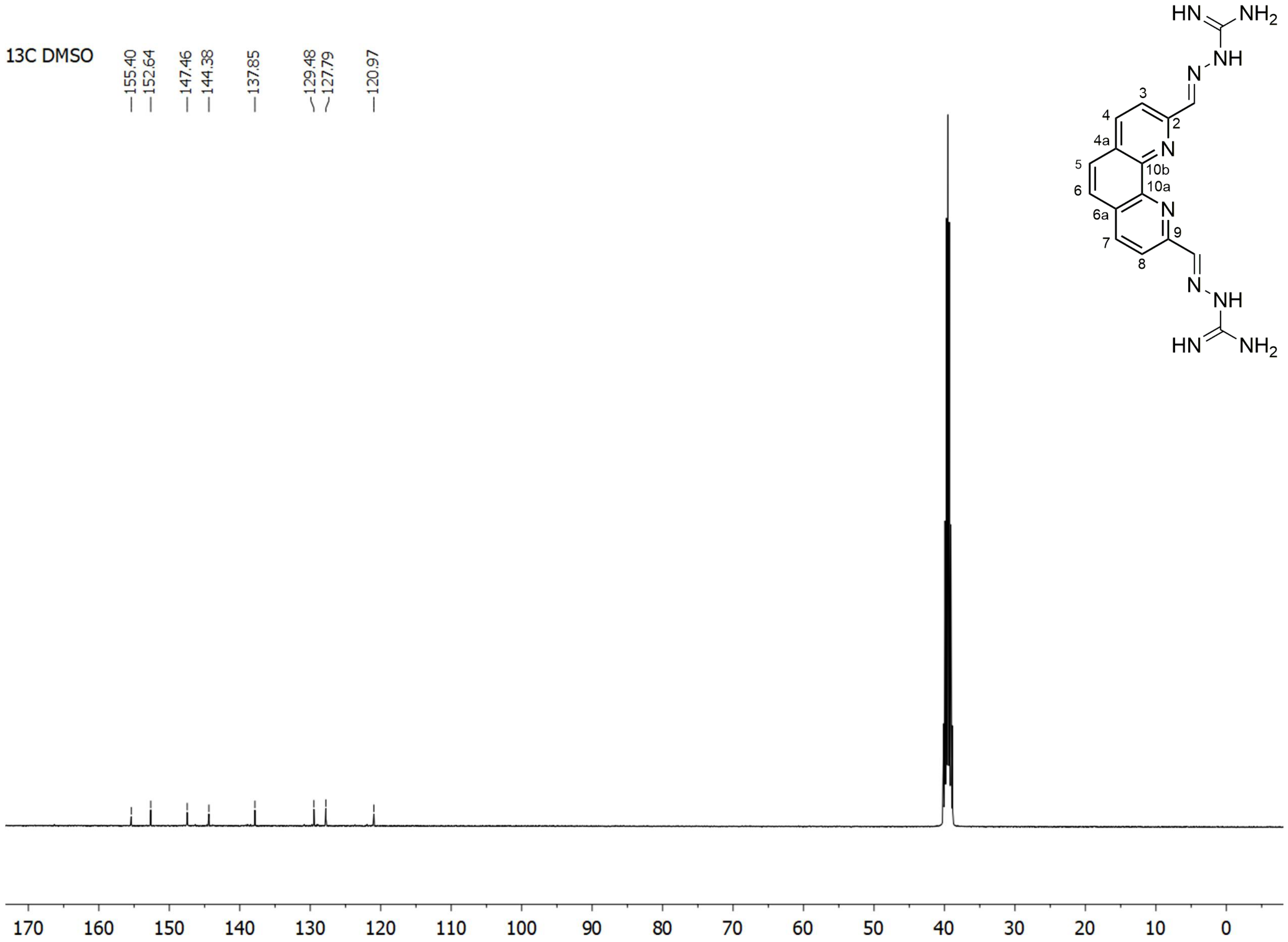

Figure S4. ^{13}C NMR (101 MHz, DMSO-d$_6$, 2.5% D$_2$O) of PhenQE8

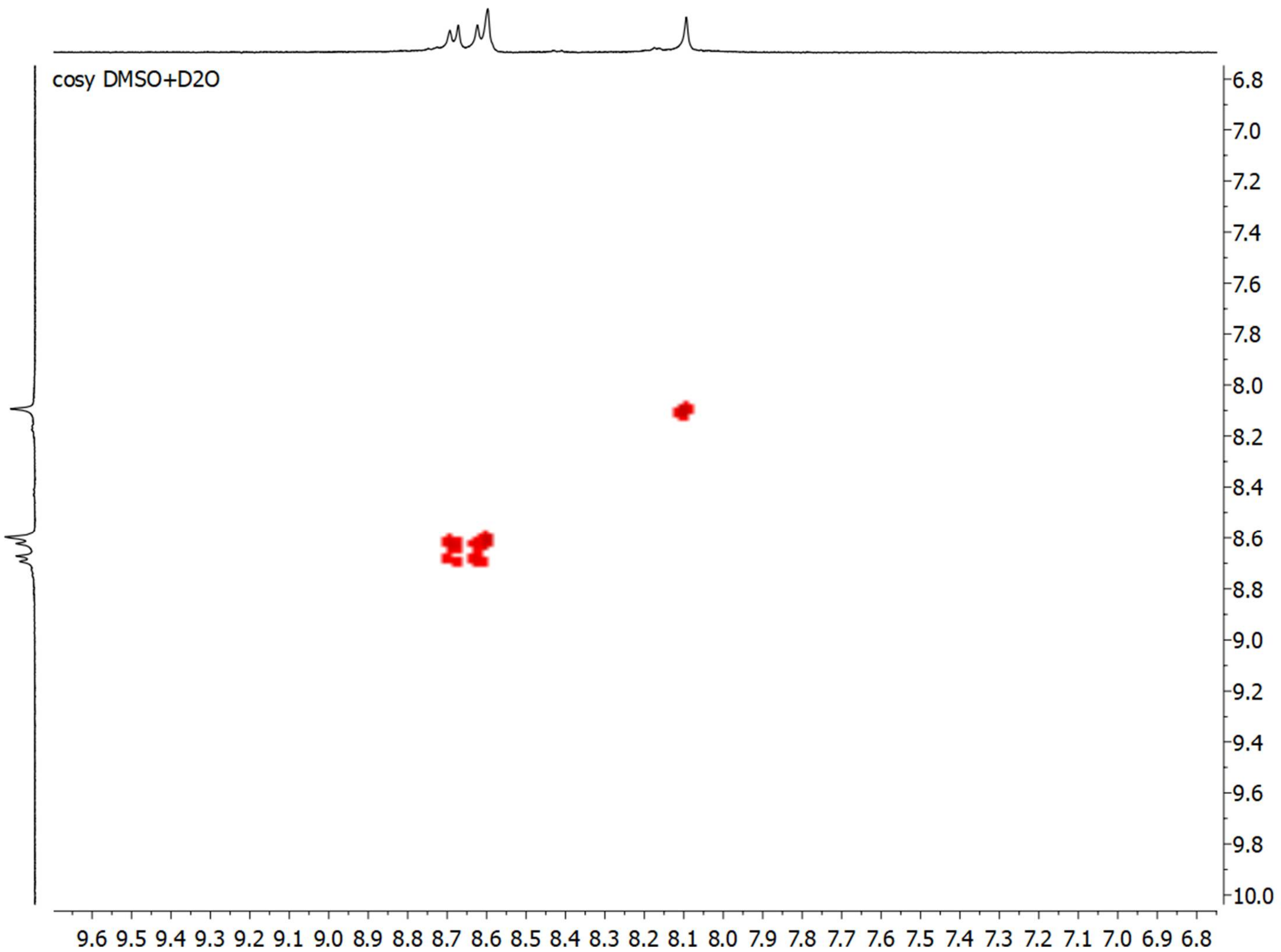

Figure S5. ^{1}H-^{1}H-COSY (400 MHz, DMSO-d_6, 2.5% D_2O) of PhenQE8

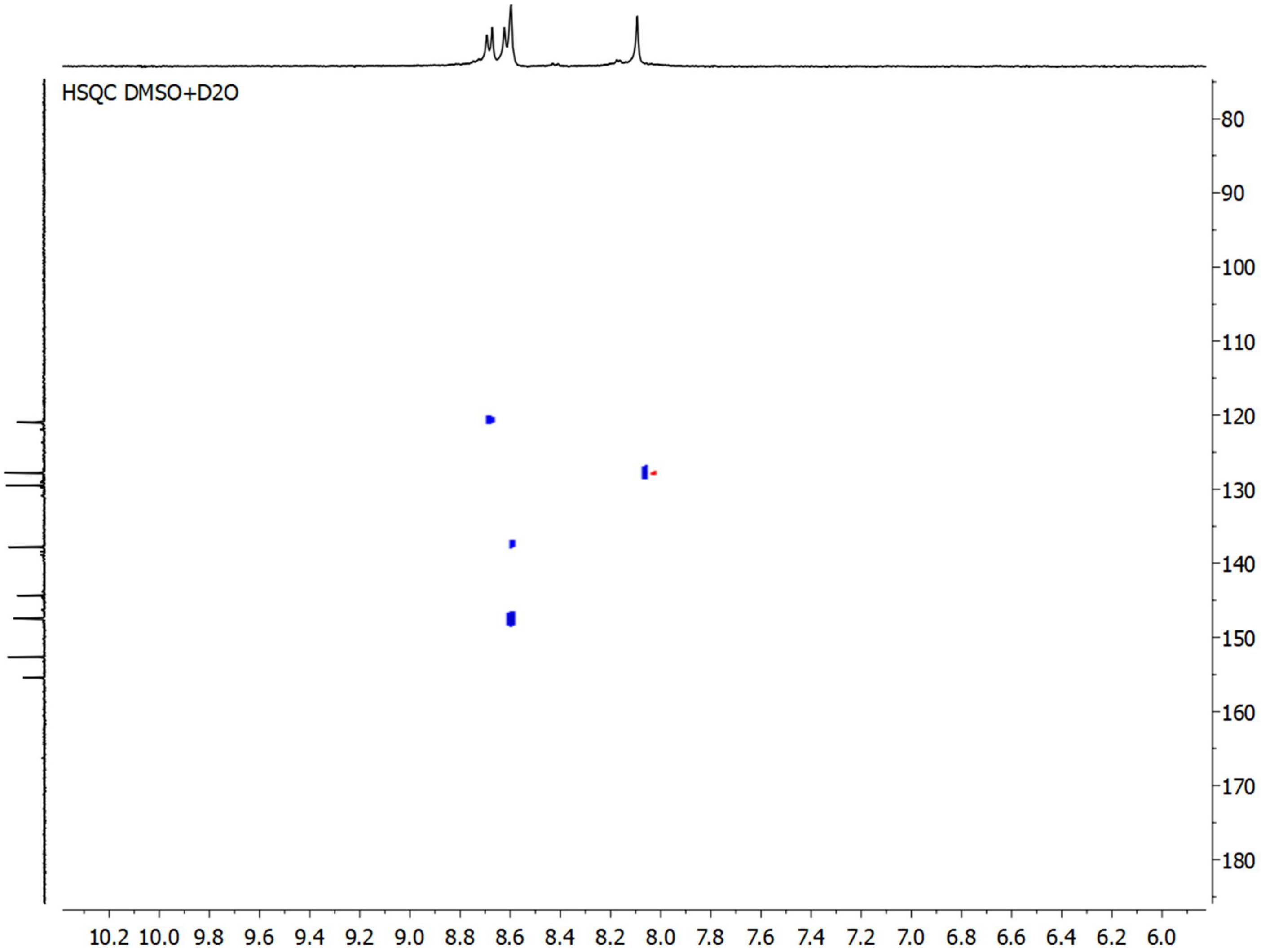

Figure S6. ^{1}H-^{13}C-HSQC (400 MHz, DMSO-d$_6$, 2.5% D_2O) of PhenQE8

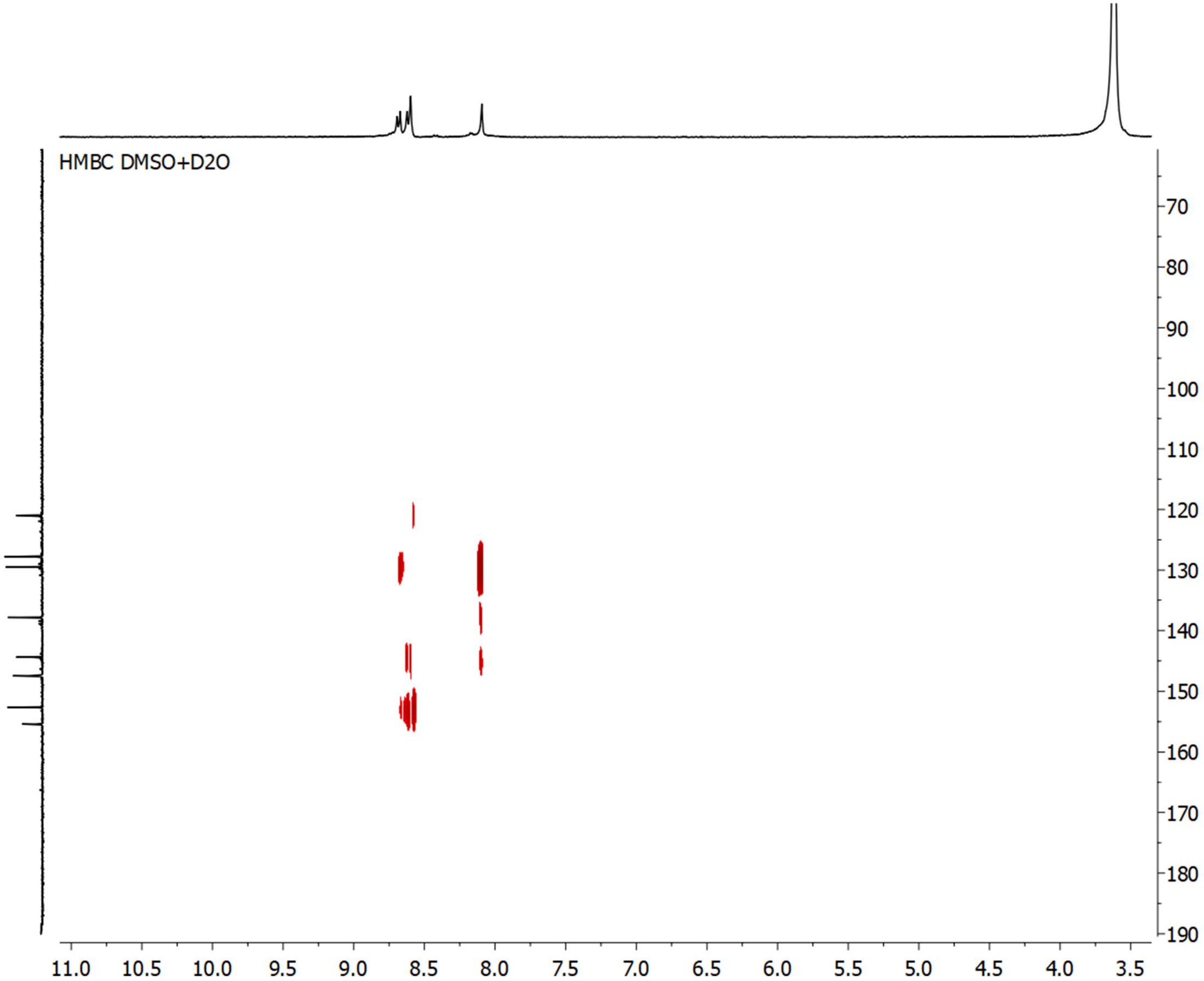

Figure S7. ^{1}H-^{13}C-HMBC (400 MHz, $(CD_3)_2SO$, 2.5% D_2O) of PhenQE8

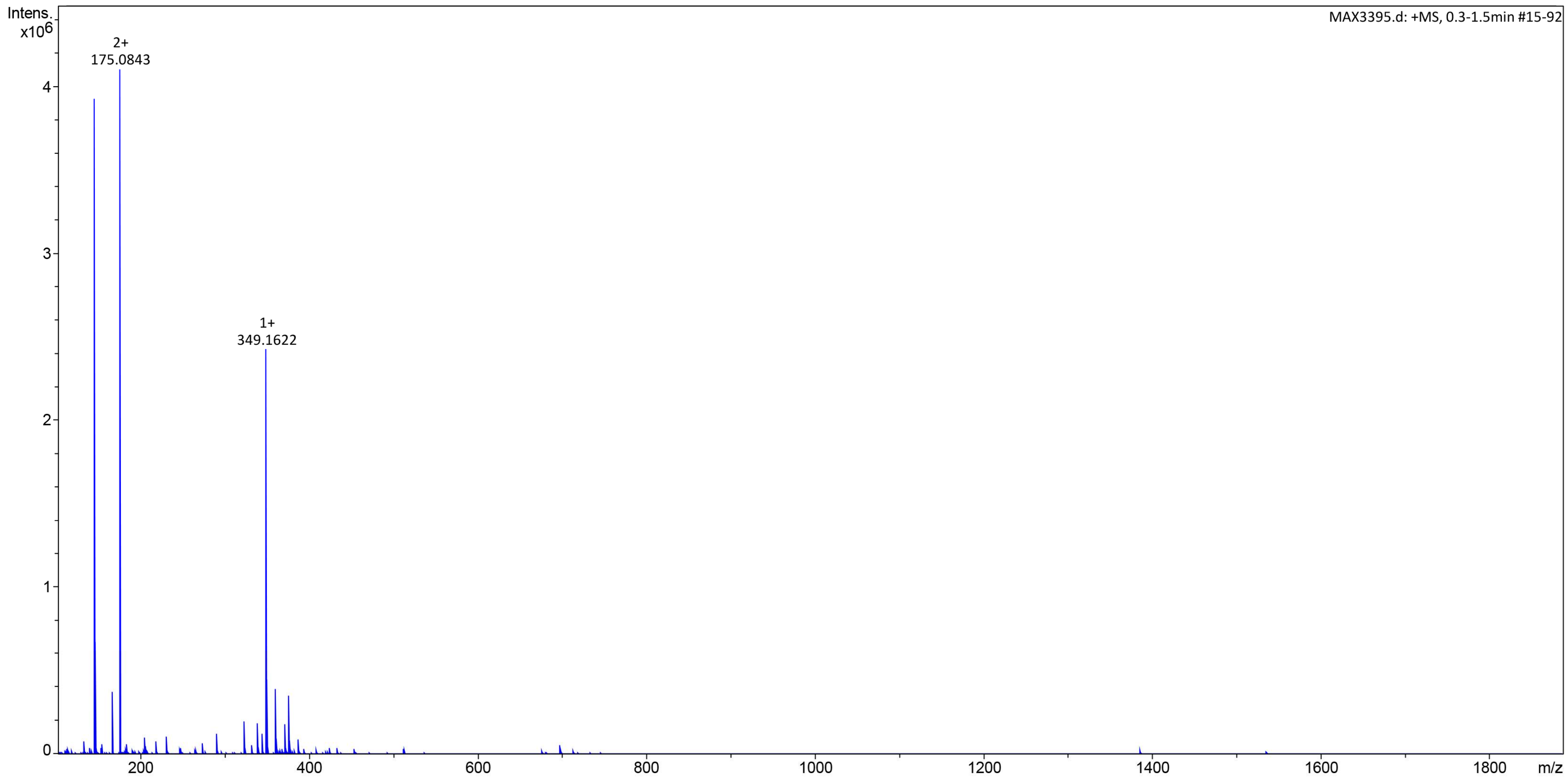

Figure S8. Full high-resolution electrospray ionization mass spectrum (ESI-HRMS) of PhenQE8 (MeOH, 1% formic acid)

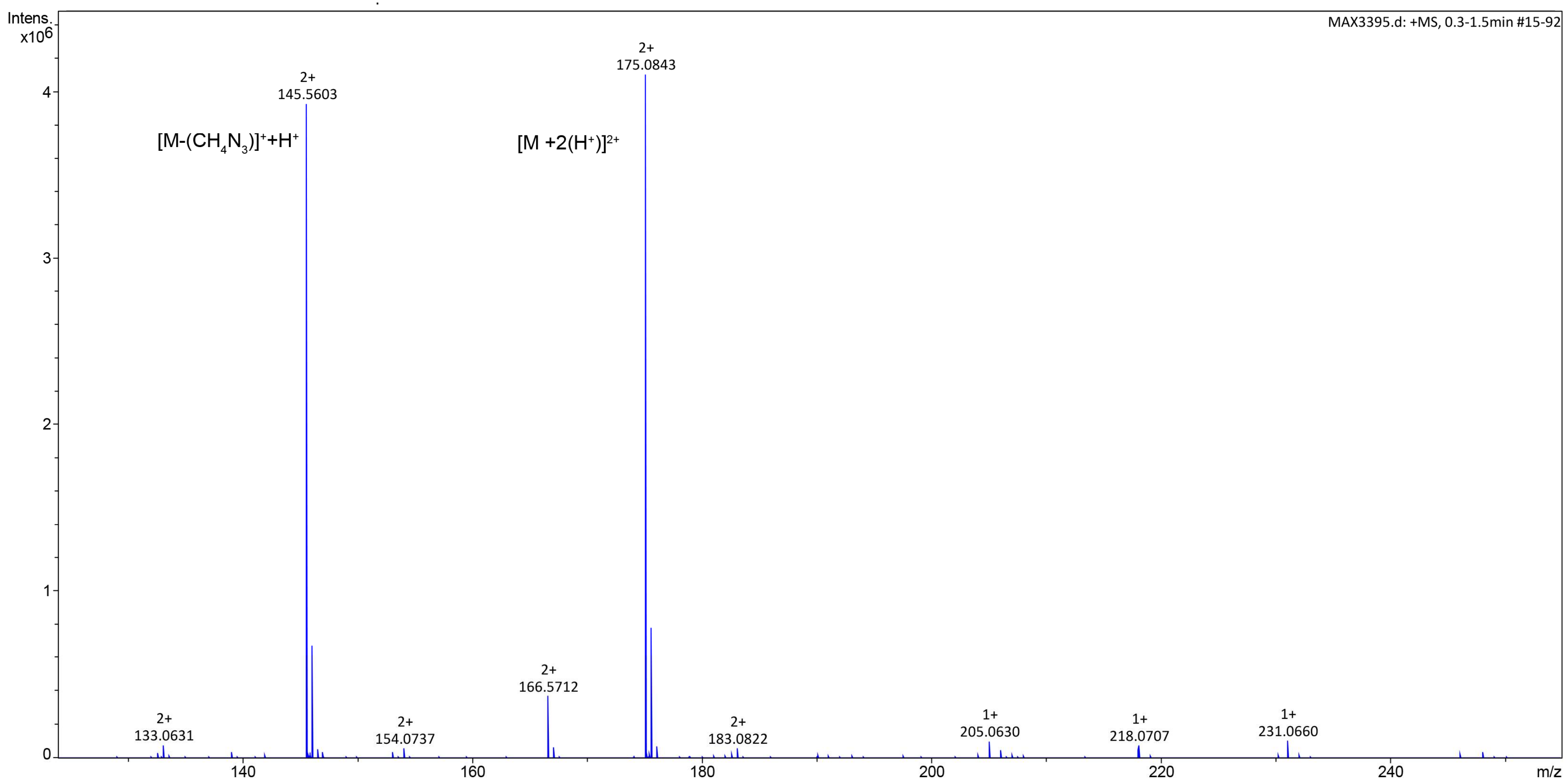

Figure S9. Zoomed view of high-resolution electrospray ionization mass spectrum (ESI-HRMS) of PhenQE8 (MeOH, 1% formic acid).

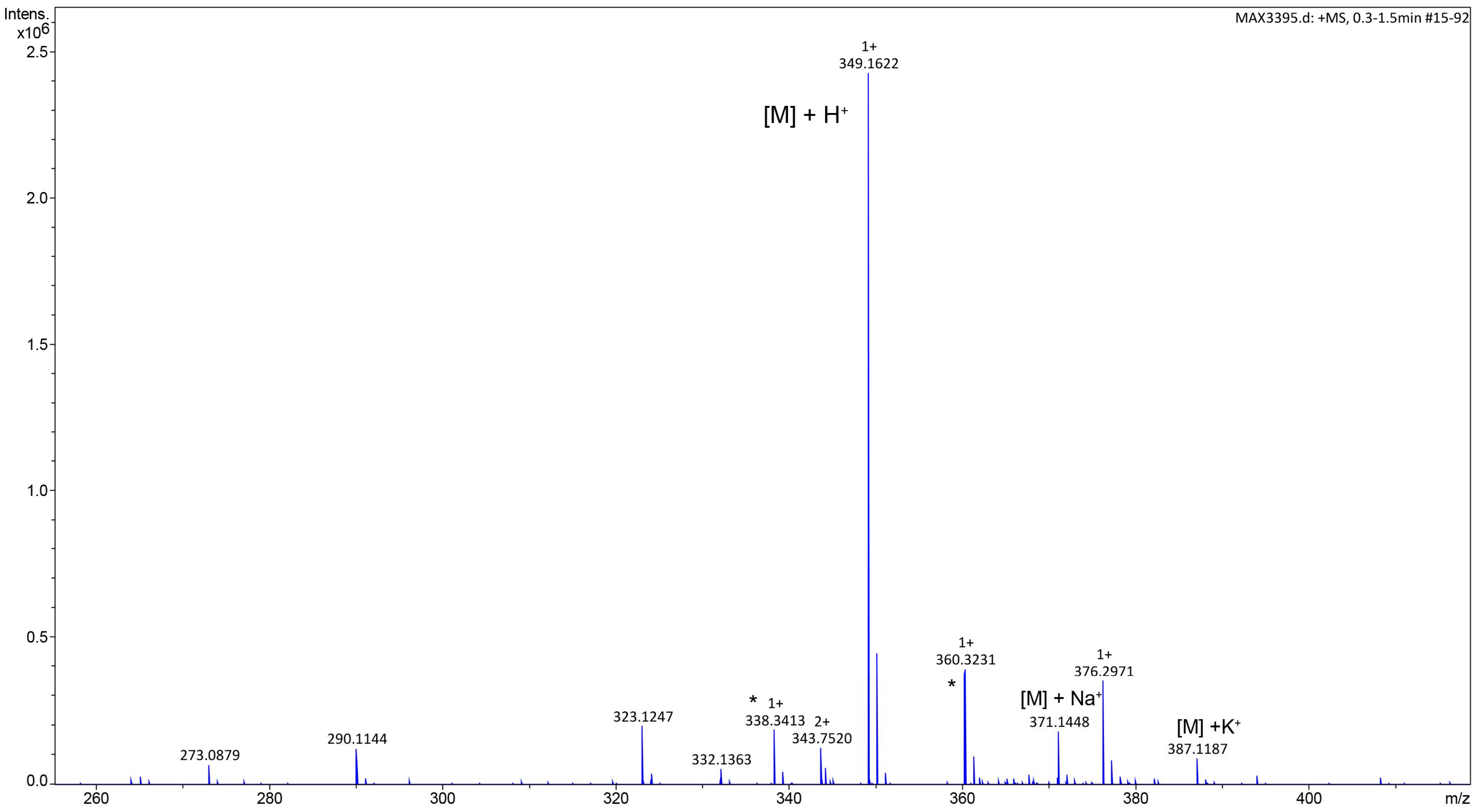

Figure S10. Zoomed view of high-resolution electrospray ionization mass spectrum (ESI-HRMS) of PhenQE8 (MeOH, 1% formic acid)

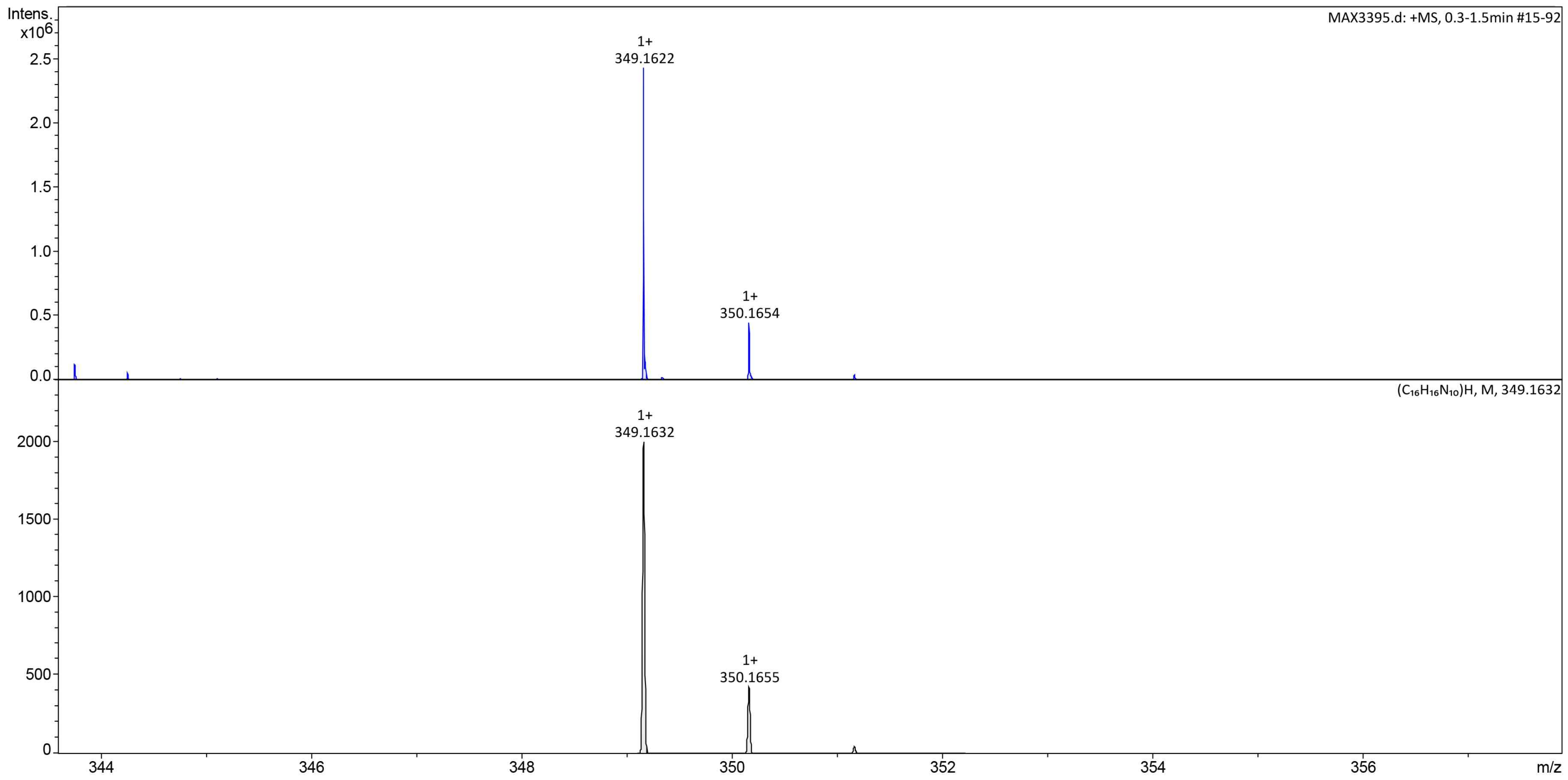

Figure S11. Zoomed view of high-resolution electrospray ionization mass spectrum (ESI-HRMS) of PhenQE8 (MeOH, 1% formic acid). Top panel represents the experimental isotopic distribution of protonated molecular ion. Bottom panel represents calculated isotopic distribution for [M] + H^+.

Table S1. Experimental data for the X-ray diffraction study on compound **PhenQE8**

formula	$C_{16}H_{16}N_{10}$
CCDC[a] code	2041050
M_r	348.39
T [K]	200(2)
λ [Å]	0.71073
crystal system	monoclinic
space group	$C2$
a [Å]	33.889(4)
b [Å]; β (º)	11.308(1); 91.50(1)
c [Å]	9.965(1)
V [Å^3]	3817.3(6)
Z	6
ρ_{calcd} [g cm^{-3}]	0.909
$\mu_{MoK\alpha}$ [mm^{-1}]	0.061
F(000)	1092
crystal size [mm^3]	0.46 × 0.22 × 0.08
θ range (deg)	3.20 to 27.50
index ranges	−44 to 44<br>−14 to 14<br>−12 to 12
reflns collected	42745
unique data	8673 [R(int) = 0.125]
obsd data [$I > 2\sigma(I)$]	6837
GOF on F^2	1.068
final R[b] indices [$I > 2\sigma(I)$]	R1 = 0.081<br>wR2 = 0.195
R[b] indices (all data)	R1 = 0.103<br>wR2 = 0.206
largest diff. peak/hole [e Å^{-3}]	0.464 and −0.348

[a] Cambridge Crystallographic Data Centre. [b] $R1 = \sum||F_0|-|F_c||/[\sum|F_0|]$; $wR2 = \{[\sum w(F_0^2 - F_c^2)^2]/[\sum w(F_0^2)^2$

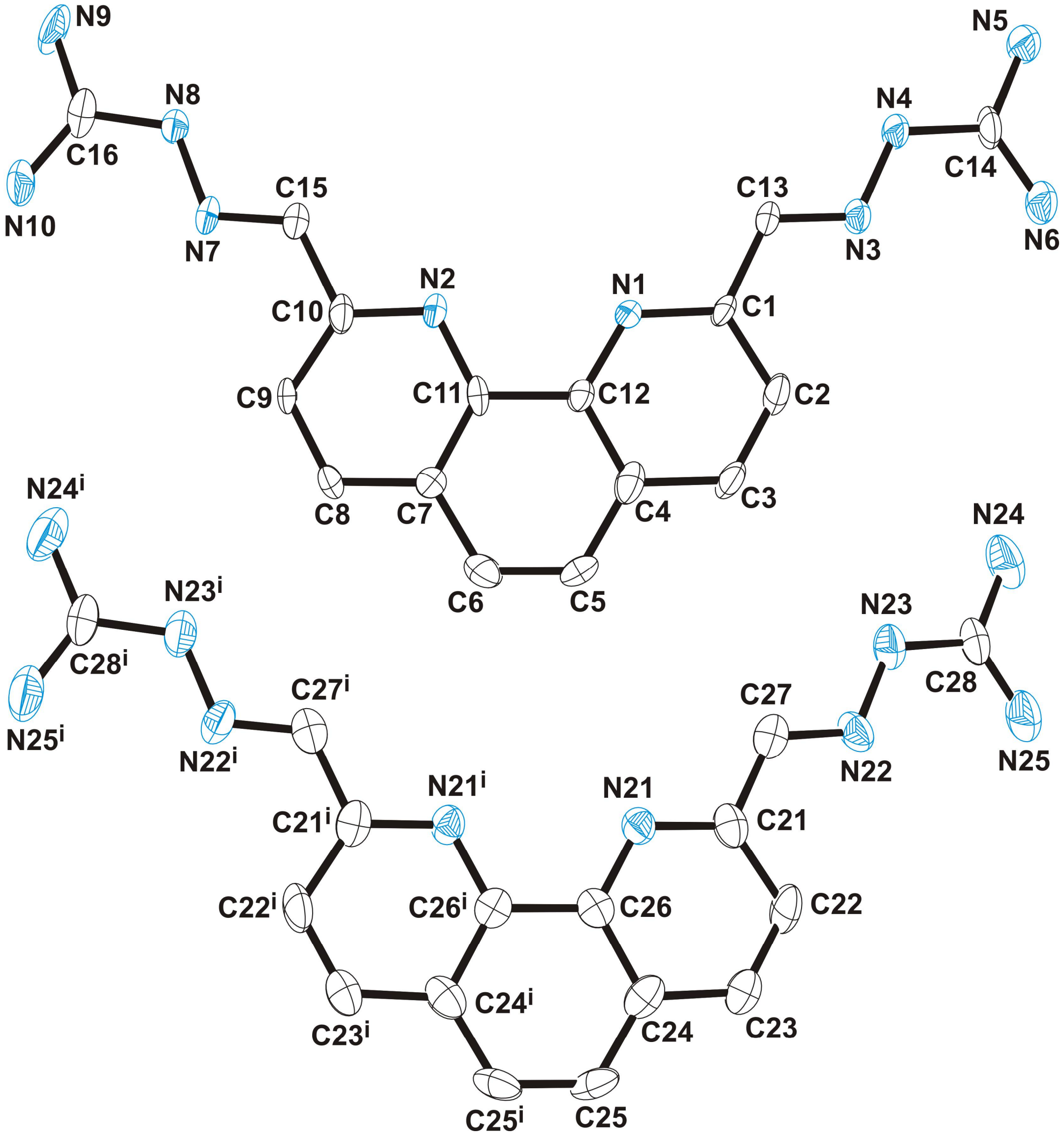

Figure S12. X-ray structure and numbering scheme for both independent molecules of PhenQE8. Thermal ellipsoids are drawn at the 50% probability level. Symmetry code: (i) $1 - x, y, -z$.

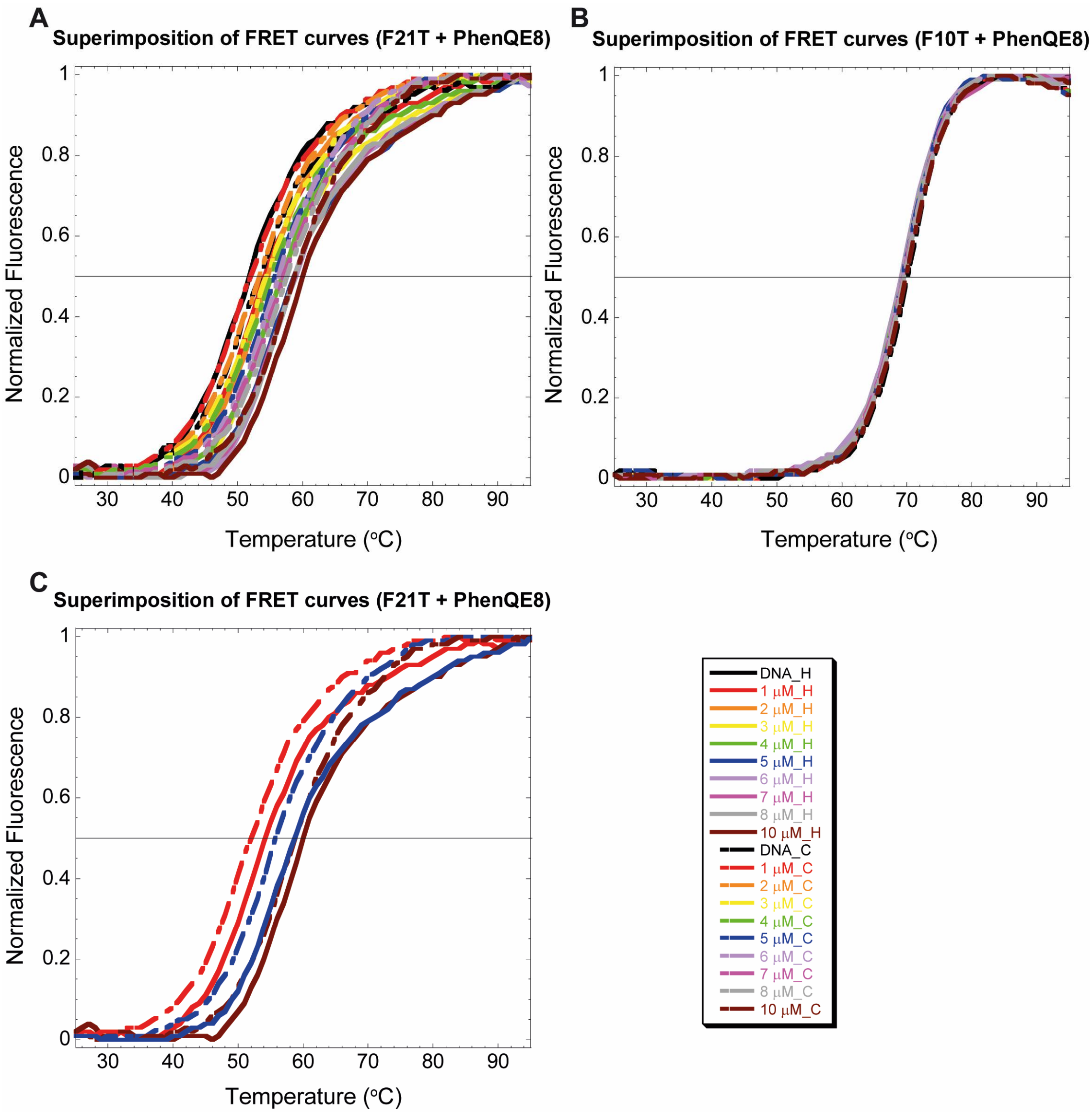

Figure S13. Superimposition of FRET heating (H) curves and cooling (C) curves corresponding to the processes of unfolding and folding of DNA, respectively. A) With Tel22 and PhenQE8 at a concentration range between 1-10 μM, in the buffer system 10 mM lithium cacodylate, 90 mM LiCl, 10 mM KCl. B) with duplex DNA F10T at a concentration range between 1-10 μM.C) With Tel22 and PhenQE8 at three separate concentration points of the titration (1μM, red; 5 μM, blue; 10 μM, brown). Solid lines represent the heating curves (H), whereas dashed lines correspond to cooling curves (C).

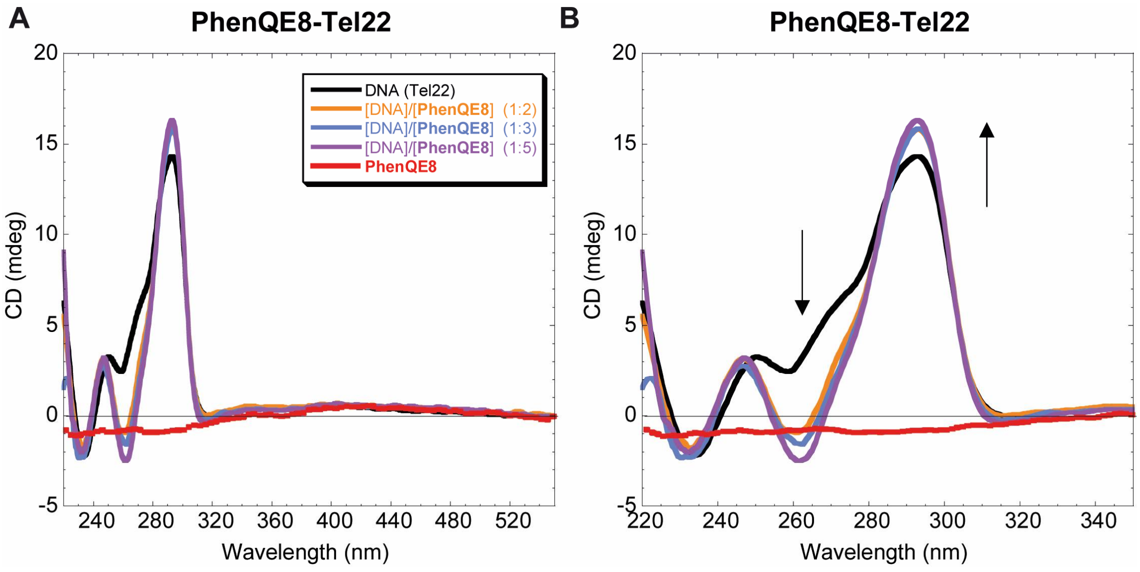

Figure S14. A) Changes in the CD spectra of quadruplex Tel22 (8 μM strand molarity, 220-350 nm region), folded in the presence of potassium ions (10 mM lithium cacodylate, 90 mM LiCl, 10 mM KCl, pH 7.3, 25 ºC) upon addition of PhenQE8 at three different quadruplex DNA/PhenQE8 molar ratios (1:2, 1:3 and 1:5). The spectrum of PhenQE8 in the same experimental conditions at 8 μM is shown. B) Zoomed view of the CD spectrum (220-350 nm).

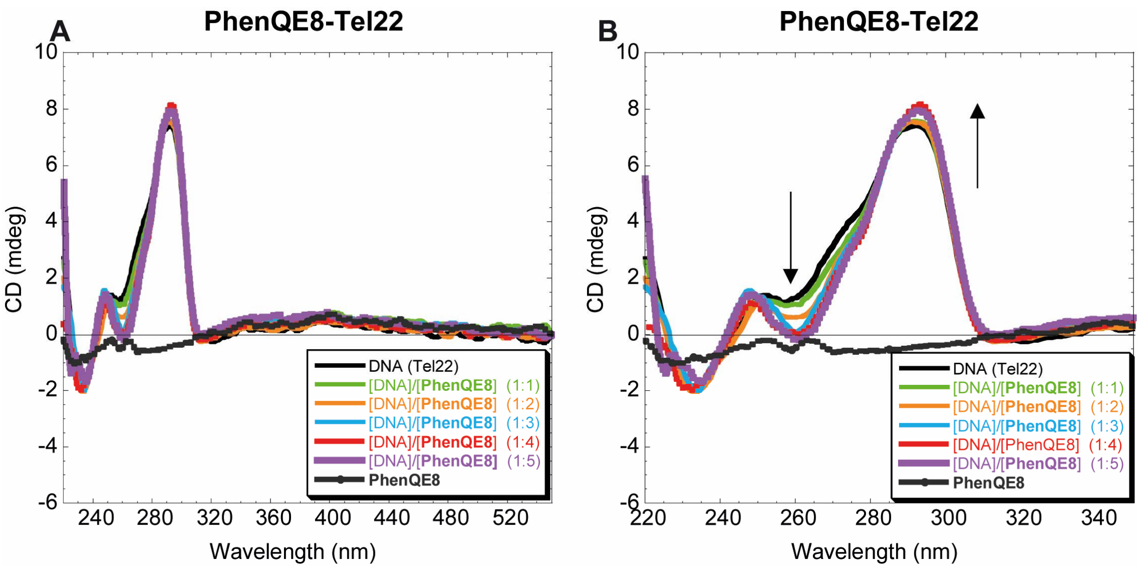

Figure S15. A) Changes in the CD spectra of quadruplex Tel22 (4 μM strand molarity, 220-350 nm region), folded in the presence of potassium ions (10 mM potassium phosphate, 100 mM KCl, pH 7.2, 25 ºC) upon addition of PhenQE8 in a molar ratio quadruplex DNA/PhenQE8 ranging from 1:1 to 1:5. The spectrum of PhenQE8 in the same experimental conditions at 8 μM is shown. B) Zoomed view of the CD spectrum (220-350 nm).

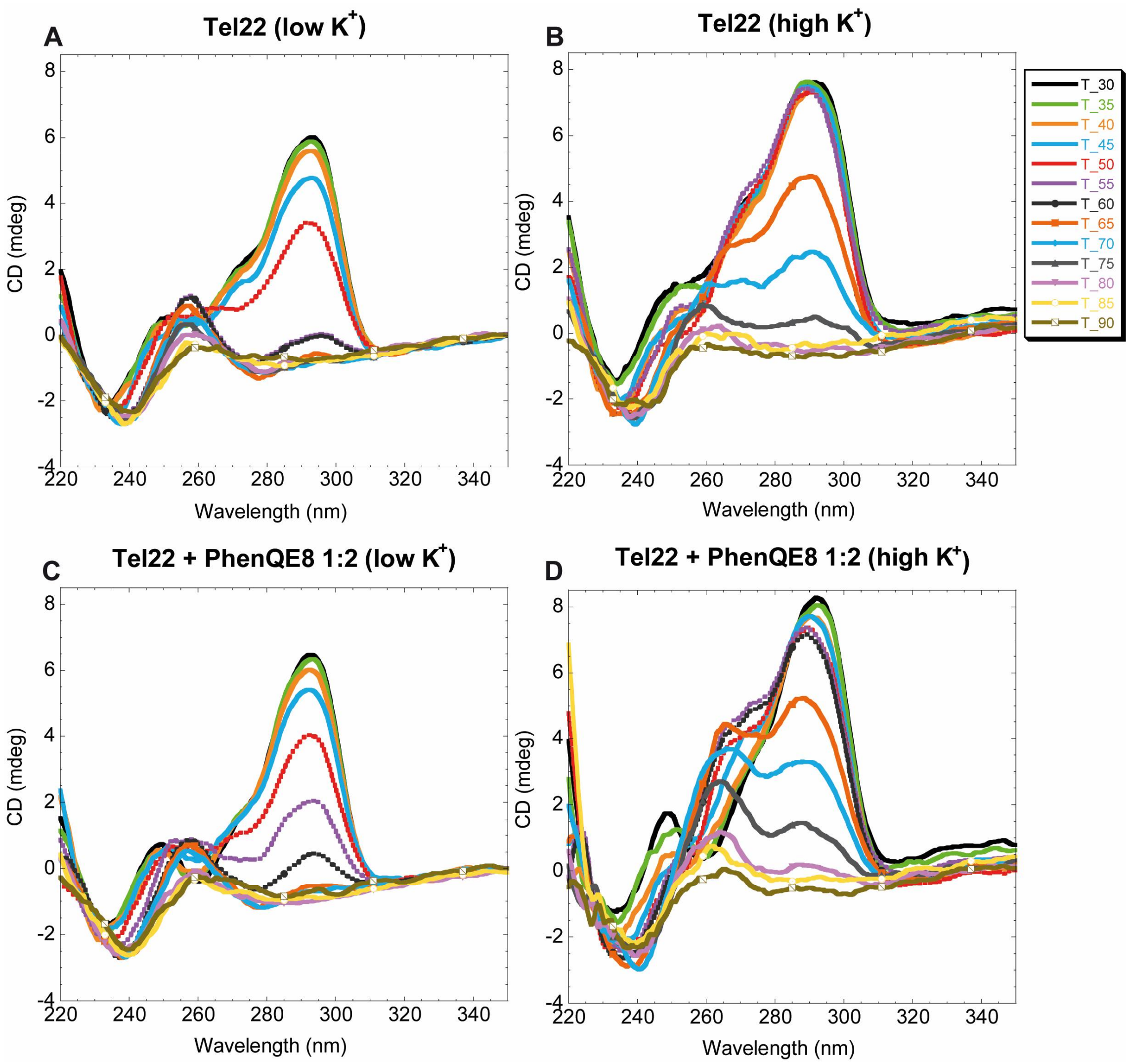

Figure S16. Unfolding CD spectra of quadruplex Tel22 (4 μM strand molarity, 220-350 nm region) by increasing temperature up to 90 ºC A) Under low K^+ salt concentration conditions ions (10 mM lithium cacodylate, 90 mM LiCl, 10 mM KCl, pH 7.3). B) Under high K^+ salt concentration conditions (10 mM potassium phosphate, 100 mM KCl, pH 7.2). C) Same unfolding process as panel A upon addition of PhenQE8 in a 1:2 quadruplex DNA/PhenQE8 molar ratio. D) Same unfolding process as panel C upon addition of PhenQE8 in a 1:2 quadruplex DNA/PhenQE8 molar ratio.

Table S2. Nucleic Acid Samples Used in Competition Dialysis Experiments[a]

Deoxyribonucleic acid	λ *(nm)*	ε *(M⁻¹·cm⁻¹)*
Tel22 (5'-AGG GTT AGG GTT AGG GTT AGGG-3')	260	228,500
ds17 (5'-CCA GTT CGT AGT AACCC-3')	260	160,900
ds17 (5'-GGG TTA CTA CGA ACTGG-3')	260	167,400

[a] ε is the molar extinction coefficient at the wavelength λ, expressed in terms of DNA molarity.

Table S3. Wavelengths and Extinction Coefficients of PhenQE8 in water and in 10 mM potassium phosphate saline buffer (pH 7.2)

Compound	λ *(nm)*	ε_{H2O} $(M^{-1}cm^{-1})$	ε_{PBS} $(M^{-1}cm^{-1})$	$\varepsilon_{PBS+Triton}$ $(M^{-1}cm^{-1})$
**PhenQE8**	311	8,385	5,185 (λ =305)	5,803 (λ =305)
	285	9,573		
